# Bidirectional Interactions between Arboviruses and the Bacterial and Viral Microbiota in Aedes aegypti and Culex quinquefasciatus

**DOI:** 10.1128/mbio.01021-22

**Published:** 2022-09-07

**Authors:** Chenyan Shi, Leen Beller, Lanjiao Wang, Ana Rosales Rosas, Lander De Coninck, Lyza Héry, Laurence Mousson, Nonito Pagès, Jeroen Raes, Leen Delang, Anubis Vega-Rúa, Anna-bella Failloux, Jelle Matthijnssens

**Affiliations:** a Department of Preventive Medicine, School of Public Health, Shenzhen University, Shenzhen, China; b KU Leuven, Department of Microbiology, Immunology and Transplantation, Rega Institute, Laboratory of Clinical and Epidemiological Virology, Laboratory of Viral Metagenomics, Leuven, Belgium; c KU Leuven, Department of Microbiology, Immunology and Transplantation, Rega Institute for Medical Research, Laboratory of Virology and Chemotherapy, Leuven, Belgium; d Laboratory of Vector Control Research, Institute Pasteur of Guadeloupe-Lieu-dit Morne Jolivière, Les Abymes, Guadeloupe, France; e Arboviruses and Insect Vectors Unit, Institut Pasteur, Paris, France; f CIRAD, UMR ASTRE, Petit-Bourg, Guadeloupe, France; g ASTRE, Univ Montpellier, CIRAD, INRA, Montpellier, France; h Department of Microbiology and Immunology, Rega Institute, KU Leuven—University of Leuven, Leuven, Belgium; i VIB Center for Microbiology, Leuven, Belgium; Boston Children's Hospital; Catholic University of America

**Keywords:** single mosquito metagenomics, arbovirus, microbiome, virome, phageome, mosquito-arbovirus-microbiota interaction

## Abstract

Mosquitoes are important vectors for many arboviruses. It is becoming increasingly clear that various symbiotic microorganisms (including bacteria and insect-specific viruses; ISVs) in mosquitoes have the potential to modulate the ability of mosquitoes to transmit arboviruses. In this study, we compared the bacteriome and virome (both eukaryotic viruses and bacteriophages) of female adult Aedes aegypti and Culex quinquefasciatus mosquitoes fed with sucrose/water, blood, or blood spiked with Zika virus (ZIKV) or West Nile virus (WNV), respectively. Furthermore, we investigated associations between the microbiota and vector competence. We show that the influence of arboviruses on the mosquito microbiome—and vice versa—is distinct for each combination of arbovirus/mosquito species. The presence of ZIKV resulted in a temporarily increased *Aedes* ISV diversity. However, this effect was distinct for different ISVs: some ISVs decreased following the blood meal (Aedes aegypti totivirus), whereas other ISVs increased only when the blood contained ZIKV (Guadeloupe mosquito virus). Also, the diversity of the *Aedes* bacteriome depended on the diet and the presence of ZIKV, with a lower diversity observed for mosquitoes receiving blood without ZIKV. In *Cx. quinquefasciatus*, some ISVs increased in WNV-infected mosquitoes (Guadeloupe Culex tymo-like virus). Particularly, the presence of Wenzhou sobemo-like virus 3 (WSLV3) was associated with the absence of infectious WNV in mosquito heads, suggesting that WSLV3 might affect vector competence for WNV. Distinct profiles of bacteriophages were identified in *Culex* mosquitoes depending on diet, despite the lack of clear changes in the bacteriome. Overall, our data demonstrate a complex three-way interaction among arboviruses, resident microbiota, and the host, which is distinct for different arbovirus–mosquito combinations. A better understanding of these interactions may lead to the identification of microbiota able to suppress the ability of arbovirus transmission to humans, and hence improved arbovirus control measures.

## INTRODUCTION

In support of the holobiont concept, increasing evidences is emerging that the interactions between the host and its complex symbiotic microbial communities have a strong influence on the host biology (e.g., evolution, immunity, and vector competence) by modifying the multipartite interaction dynamics (1, 2). Some symbiotic bacteria and insect-specific viruses (ISVs), like *Wolbachia* (3, 4), *Chromobacterium* (*Csp_P*) (5), Pseudomonas rhodesiae (6), Enterobacter ludwigii (6), Serratia odorifera (7), Serratia marcescens (8), Culex flavivirus (9, 10), Palm Creek virus (11) and La Crosse virus (12), have been reported to alter mosquito susceptibility to certain arboviruses. Several studies have highlighted the abundance and diversity of ISVs and bacteria in mosquito populations (13, 14); hence, the potential vector competence affecting symbiotic interactions might not result from a single virus or bacterium independently. However, no studies so far have investigated vector competence alteration from the perspective of the entire microbial community in mosquitoes.

Using single mosquito viral metagenomics, we previously described that *Aedes* (*Ae.*) *aegypti* and *Culex* (*Cx*.) *quinquefasciatus* from Guadeloupe contain distinct and stable core eukaryotic viromes (15), and some of these core eukaryotic viruses are conserved across different *Aedes* mosquito species from different countries and continents (16). These core eukaryotic viruses are likely to be ISVs, which live in a subtle equilibrium with the host immune system. Among the core viruses, Phasi Charoen-like phasivirus (PCLPV), together with cell fusing agent virus, could strongly inhibit the growth of Zika virus (ZIKV) and dengue virus in Aa23 cell lines (12). Therefore, the influence of those ISVs on the transmission of pathogenic arboviruses, or vice versa, is worth further investigation. Additionally, viral quantification obtained in a previous study (15) showed that the viral load of the core ISVs is variable among different individual mosquitoes of the same species. The reason for this large observed variation is currently unknown but might be immune related and could therefore be important for a better understanding of vector competence. Furthermore, the bacteriophage component (infecting bacteria) is generally neglected in mosquito virome research. Their influence on shaping the structure of bacterial microbiota in mosquitoes remains elusive.

Guadeloupe experienced a serious ZIKV outbreak in 2016, with 30,500 suspected clinical cases (17), and *Ae. aegypti* has been shown to be the main vector of ZIKV (18, 19). WNV seroconversions in Guadeloupe have been detected several times in horses and birds since 2002, but no human cases have been reported so far (20). Although the vector competence of *Culex* spp. mosquitoes from Guadeloupe has never been evaluated for WNV, *Cx. quinquefasciatus* is the most abundant *Culex* mosquito species in urban settings (21) and has the potential to transmit this virus (10).

In this study, we allowed eggs of *Ae. aegypti* and *Cx. quinquefasciatus* collected in the field from Guadeloupe in 2018 to hatch in the lab. Female *Ae. aegypti* and *Cx. quinquefasciatus* adults were fed with sucrose/water, blood, or blood spiked with ZIKV or West Nile virus (WNV), respectively. Both the bacteriome and virome (both eukaryotic viruses and bacteriophages) in individual mosquitoes of these three groups were analyzed.

After ingestion of a blood meal from a viraemic vertebrate host, arbovirus transmission to the next vertebrate hosts requires a successful viral infection and replication in the mosquito midgut and salivary glands (22 to 24). Such a dissemination includes several critical steps: (i) invasion and establishment of infection in the midgut epithelial cells; (ii) dissemination of the virus from the midgut to the haemocoel and secondary organs, including the salivary glands, and (iii) successful replication in the salivary glands and expectoration in saliva during biting (24 [Bibr B25]to 26). Therefore, in this study, the heads of individual mosquitoes engorged with spiked blood were titrated by plaque assays to determine the presence of infectious ZIKV or WNV viral particles, as a proxy for the ability to transmit the virus during a next bite on a mammalian or avian host. The study design is summarized in Fig. S1 in the supplemental materials.

10.1128/mbio.01021-22.2 FIG S1 Flowchart of study design. Female adults, which hatched in the lab from eggs of *Ae. aegypti* and *Cx. quinquefasciatus* collected in the field from Guadeloupe in 2018. The 7-day-old female adults were fed with one of three meals: (a) sucrose and water; (b) regular blood; or (c) blood spiked with infectious ZIKV (*Ae. aegypti*) or WNV (*Cx. quinquefasciatus*). Individual mosquitoes from all three groups were sacrificed at 7 and 21 (ZIKV) or 14 (WNV) days postexposure (dpe). The heads of individual mosquitoes engorged with spiked blood were titrated by plaque assays to determine the presence of infectious ZIKV or WNV viral particles. Viral metagenomics and 16S rRNA sequencing were applied on the bodies of all the mosquitoes to explore the associations of the mosquito microbiome with arbovirus transmission. Note: No qRT-PCR was performed for WNV on the heads of Culex quinquefasciatus mosquitoes, due to insufficient sample. Download FIG S1, PDF file, 1.3 MB. Copyright © 2022 Shi et al. 2022 Shi et al. https://creativecommons.org/licenses/by/4.0/ This content is distributed under the terms of the Creative Commons Attribution 4.0 International license.

## RESULTS

### ZIKV and WNV, respectively, disseminated in *Ae. aegypti* and *Cx. quinquefasciatus* from Guadeloupe.

Infectious ZIKV particles in *Ae. aegypti* heads were only detected at 21 days postexposure (dpe) with 12.1% (5/31) dissemination efficiency (Table 1). Titers of three heads ranged from 2.7 × 10^2^ to 3 × 10^4^ PFU, and two heads only showed cytopathogenic effect (CPE), without clear plaques (Data Set S1). In *Cx*. *quinquefasciatus*, dissemination of WNV was observed at 7 and 14 dpe, both with 25% (5/20) dissemination efficiency. The WNV load per head ranged from 2 to 66.7 PFU for two samples at 7 dpi and two at 14 dpi. Three samples at 7 dpi and three at 14 dpi had only CPE. WNV showed a higher dissemination efficiency in *Cx. quinquefasciatus* compared to ZIKV in *Ae. aegypti*, but the viral titer of WNV was lower than ZIKV (Wilcoxon test, *P* value < 0.0001).

**TABLE 1:** Dissemination efficiencies of ZIKV and WNV

Mosquito species	Viral strain	Day postexposure	Dissemination efficiency	Titer (PFU per head)
Aedes aegypti	ZIKV Martinique strain (GenBank: KU647676)	7	0% (0/33)	
		21	12.1% (5/41)	2.7 × 10^2^ to 3 × 10^4^
Culex quinquefasciatus	WNV from Camargue (France) (GenBank: AY268132)	7	25% (5/20)	2 to 66
		14	25% (5/20)	

10.1128/mbio.01021-22.7 DATA SET S1 Eukaryotic virome in *Aedes* and *Culex* mosquitoes. Download Data Set S1, XLSX file, 0.1 MB. Copyright © 2022 Shi et al. 2022 Shi et al. https://creativecommons.org/licenses/by/4.0/ This content is distributed under the terms of the Creative Commons Attribution 4.0 International license.

### Virome identification.

The 154 mosquito bodies (excluding head, legs, and wings) were individually processed for virome analyses (Table 2). The clustering of assembled contigs from all samples longer than 1 kb based on 95% nucleotide identity over 80% of the length resulted in 133,951 representative contigs (Data Set S1). After removing the viral contigs presented in both negative control samples, 127 of the remaining contigs were annotated as eukaryotic viruses by DIAMOND, BLASTn, and CAT. In total, 263 contigs were annotated as phage, of which 41 could be annotated at the family level. Besides these viral contigs, nearly half of the representative contigs belonged to mosquito genomes and 9.3% were suspected bacterial contigs. In *Ae*. *aegypti* and *Cx*. *quinquefasciatus* samples, 353 million and 163 million reads were mapped to all the nonredundant contigs, respectively. The eukaryotic viral reads and phage reads occupied 5% and 0.05% in *Ae*. *aegypti* samples, and 3.3% and 6.7% in *Cx*. *quinquefasciatus* samples, respectively.

**TABLE 2:** Numbers of mosquito body samples used for NGS

Mosquito species	Day postexposure	Virus/blood^*a*^	Blood	Sucrose/H2O	Total no.
Aedes aegypti	7	33	5	5	43
	21	41	5	5	51
Culex quinquefasciatus	7	20	5	5	30
	14	20	5	5	30

a To maximize the statistical power of our analyses, we selected all available engorged mosquitoes, explaining the uneven distribution of included mosquitoes.

### Increased eukaryotic virome richness in *Ae. aegypti* after receiving a ZIKV-spiked blood meal at 7 dpe.

**FIG 1:**
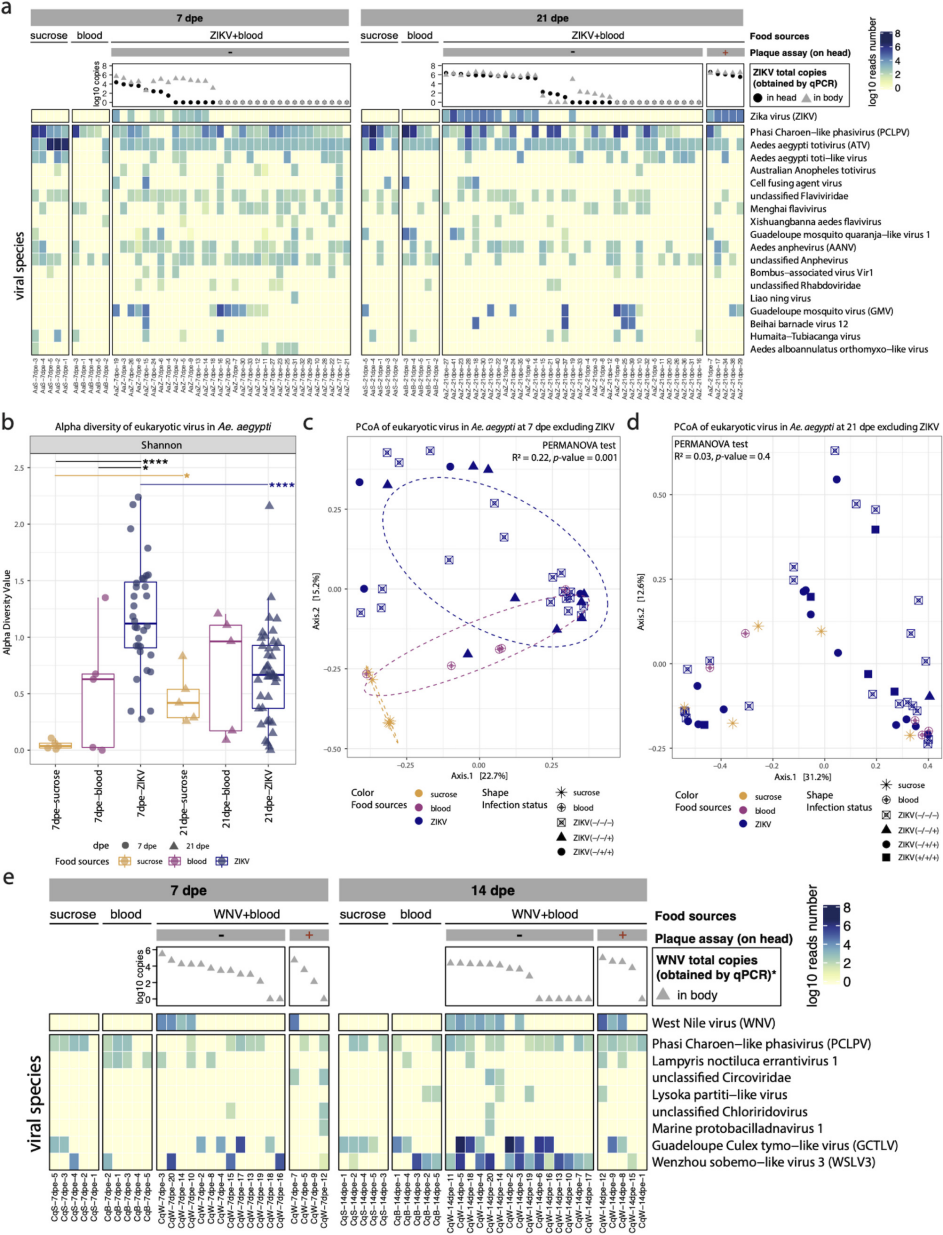
Eukaryotic virome profile in Aedes aegypti and Culex quinquefasciatus. (a) Abundance of eukaryotic viral species in Aedes aegypti. The plaque assay results are indicated by “+” for positive results and “–” for negative results in the gray bars. The dot plots show the total ZIKV genome copies in Aedes aegypti heads and bodies determined by qRT-PCR. The heatmap shows the read counts on log_10_ scale. The viral species names shown in the heatmap are from the taxonomic annotation by DIAMOND and KronaTools. The heatmap only shows the viral species and samples that had more than 500 eukaryotic viral reads (21 viral species and two samples were removed). (b) Alpha diversity of eukaryotic viral species in Aedes aegypti at 7 and 21 dpe. Pairwise Wilcoxon test: *P < *0.05 (*), *P < *0.01 (**), *P < *0.001 (***), *P < *0.0001 (****). *P* values were corrected for multiple comparisons using the Benjamini-Hochberg method. (c and d) PCoA of eukaryotic viral species in Aedes aegypti based on Bray-Curtis dissimilarity. The PERMANOVA test was performed on food sources. The “+” or “–” in the bracket after ZIKV indicates the positive or negative result of plaque assay (head), qRT-PCR detection of ZIKV in head and body, respectively. (e) Abundance of eukaryotic viral species in Culex quinquefasciatus. The plaque assay results are indicated by “+” for positive results and “–” for negative results in the gray bars. The dot plots show the total WNV genome copies in Culex quinquefasciatus heads and bodies determined by qRT-PCR. The heatmap shows the reads counts on log_10_ scale. The viral species names shown in the heatmap are from the taxonomic annotation by DIAMOND and KronaTools. Three samples with zero eukaryotic viral reads and 16 species containing less than 500 reads were deleted from the heatmap. Note: No qRT-PCR was performed for WNV on the heads of Culex quinquefasciatus mosquitoes, due to insufficient sample.

The heatmap in Fig. 1a displays the abundance of 18 eukaryotic viral species (which have more than 500 reads in total) in each sample. The five *Aedes* mosquitoes with infectious ZIKV in their heads at 21 dpe displayed high levels of ZIKV nucleic acid in both their heads and bodies detected by qRT-PCR and viral metagenomics (Fig. 1a). Several mosquitoes without measurable infectious viral particles in the head, however, contained a high number of ZIKV genome copies in their bodies and heads (as measured by qRT-PCR), as well as a high number of ZIKV reads in their bodies from next-generation sequencing (NGS) analyses. The previously identified core viruses—PCLPV, Aedes aegypti totivirus (ATV), Guadeloupe mosquito virus (GMV), and Aedes aegypti anphevirus (AANV) (15), were detected in 75.5%, 78.7%, 23.4%, and 34% of *Ae. aegypti* mosquitoes in this study (across different conditions), respectively. GMV was not detected in mosquitoes fed on noninfectious blood (both at 7 and 21 dpe), whereas several mosquitoes in the ZIKV-spiked blood group (both at 7 and 21 dpe) showed a high abundance of GMV reads. In contrast, at 7dpe, mosquitoes fed with sucrose/water showed extremely high abundance (ranging from 366,558 to 3.5 million reads) of ATV, whereas this virus was nearly absent in regular blood-fed mosquitoes, and only present at low levels in the ZIKV-spiked blood group.

For further analyses, the *Ae. aegypti* mosquitoes that received a ZIKV-spiked blood meal were separated into groups depending on their plaque assay results (positive or negative) and ZIKV genome copy numbers in the head and body (using 1,000 copies as threshold). First, the alpha diversity (Shannon index) of eukaryotic viral species was compared between the sample collection time points and different diet groups (Fig. 1b). At 7 dpe, the *Ae. aegypti* group receiving a ZIKV-spiked blood meal showed a significantly higher alpha diversity compared to the blood and sucrose group. This observed high alpha diversity in the ZIKV-spiked blood group was mainly driven by the mosquitoes without ZIKV detected in their head by plaque assay or qPCR (Fig. S2). No significant difference was observed between the different sample groups collected at 21 dpe (Kruskal-Wallis, *P* value = 0.53). Comparison of the two time points within the same diet group showed that the alpha diversity of the ZIKV-spiked blood group decreased over time, whereas the alpha diversity in the sucrose-fed group increased over time (Fig. 1b).

10.1128/mbio.01021-22.3 FIG S2 The alpha diversity of eukaryotic virus in Aedes aegypti of different time points and infection status. Download FIG S2, PDF file, 0.2 MB. Copyright © 2022 Shi et al. 2022 Shi et al. https://creativecommons.org/licenses/by/4.0/ This content is distributed under the terms of the Creative Commons Attribution 4.0 International license.

Bray-Curtis dissimilarities were calculated from the abundance of eukaryotic viral species (excluding ZIKV) to perform a principal coordinates analysis (PCoA) (Fig. 1c and d). The samples from mosquitoes fed with ZIKV-spiked blood and sucrose/water at 7 dpe clustered separately, whereas the samples from the blood-fed group mainly overlapped with the ZIKV-spiked blood samples with significant *P* value (0.001) in a permutational multivariate analysis of variance (PERMANOVA) test on food sources (R^2^ = 0.22) (Fig. 1c). There was no statistical difference found within the group that received ZIKV-spiked blood, based on the presence of infectious viruses in the head as determined by plaque assay. At 21 dpe, no significant difference was observed among any of the diet groups (PERMANOVA test on food sources, *P* value = 0.4, R^2^ = 0.03) (Fig. 1d).

### Near complete absence of Wenzhou sobemo-like virus 3 in Culex quinquefasciatus mosquitoes disseminating WNV.

In general, *Cx*. *quinquefasciatus* samples contained fewer eukaryotic viral reads and fewer viral species compared to *Ae*. *aegypti* samples (Fig. 1a versus 1e), which is consistent with the results of our previous study (15). In 22.2% (2/9) of the mosquito bodies that were WNV plaque-assay positive, no WNV could be detected by NGS and qRT-PCR (Fig. 1e). In the samples without infectious viral particles in the head (*n* = 28), 11 at 7 dpe and 9 at 14 dpe harbored at least 10^2^ WNV genome copies in their bodies (Fig. 1e). Among the previously identified core viruses of *Cx*. *quinquefasciatus* (15), PCLPV and Guadeloupe Culex tymo-like virus (GCTLV) were found in 58% and 17% of the samples, respectively (Fig. 1e), whereas GMV and Guadeloupe Culex rhabdovirus were only identified in two (with low abundance) and zero samples, respectively (Data Set S1). Notably, Wenzhou sobemo-like virus 3 (WSLV3), which was not identified in our previous study, showed high abundance in 54% of the mosquitoes fed with WNV-spiked blood without infectious WNV in their heads at 14 dpe, whereas this virus was absent in the plaque assay positive samples (except for one sample with only 80 reads). Due to the relatively low number of viral species in *Cx. quinquefasciatus*, no alpha and beta diversity analyses were performed.

### Presence of arboviruses in blood meal modulates viral loads of specific ISVs, and vice versa.

**FIG 2:**
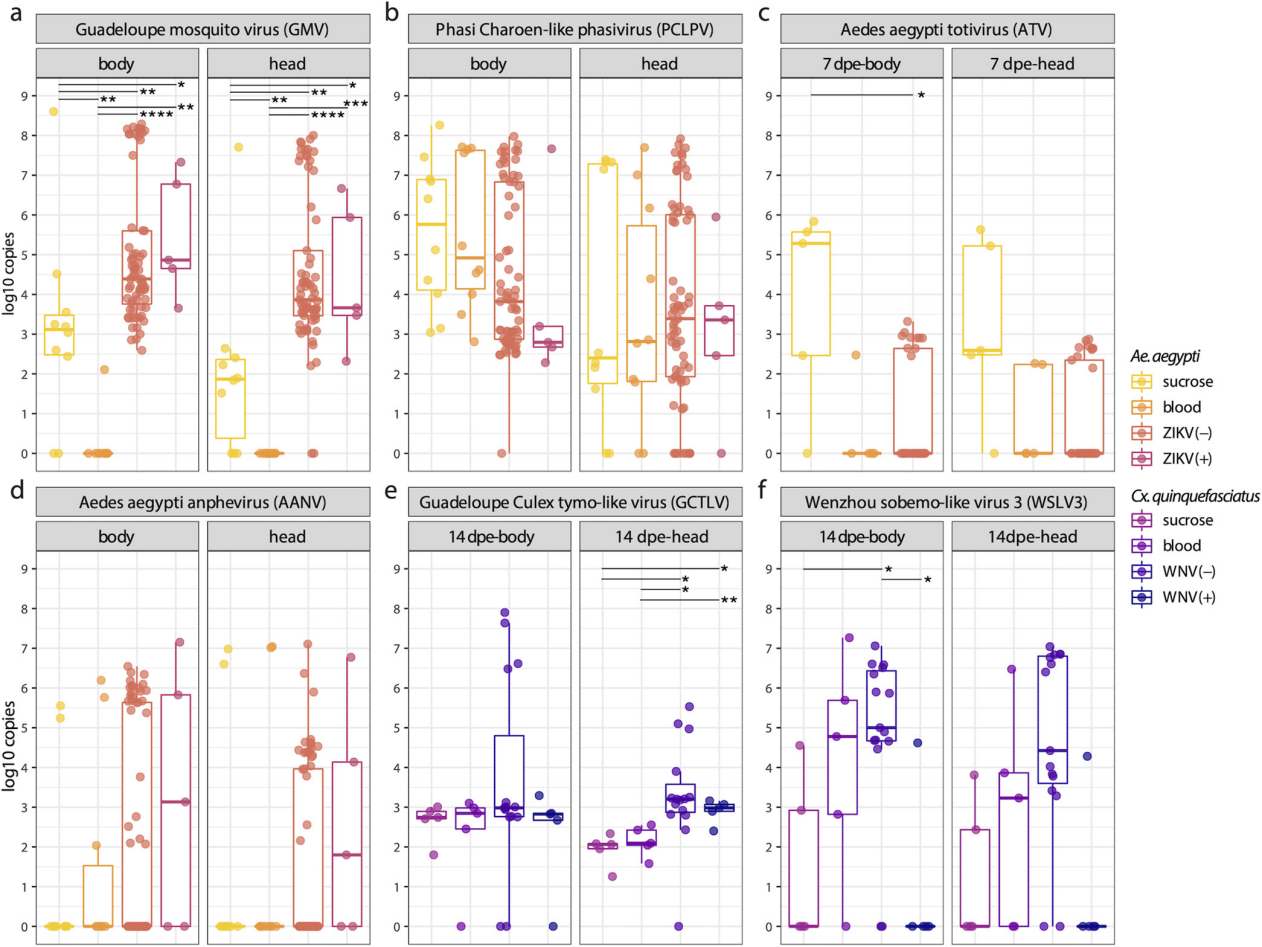
Comparison of viral genome copies in body and head mosquitoes. Pairwise Wilcoxon test: *P < *0.05 (*), *P < *0.01 (**), *P < *0.001 (***), *P < *0.0001 (****). *P* values were corrected for multiple comparisons with the Benjamini–Hochberg method. The “+” or “–” in the bracket after indicates the positive or negative result of plaque assay (head).

Since metagenomics is less sensitive compared to targeted PCR assays, and the abundance data from viral metagenomics are not fully quantitative, the genome copies of selected core viruses in the head and body of mosquitoes were further determined by qRT-PCR. The targeted ISVs were GMV, PCLPV, ATV, and AANV in *Ae*. *aegypti* (Fig. 2a to d), and GCTLV and WSLV3 in *Cx*. *quinquefasciatus* (Fig. 2e and f). Interestingly, the number of GMV genome copies was much higher in the body and head of the mosquito receiving ZIKV-spiked blood (with median around 10^4^ copies) compared to blood meal without ZIKV, or the sucrose meal. The virus is almost absent in the mosquitoes that received a noninfectious blood meal (except for one sample with about 100 genome copies) (Fig. 2a). There was no significant difference of PCLPV genome copies among different groups or time points (Fig. 2b and Fig. S3). As observed in Fig. 1a, the viral genome copy number of ATV in the bodies of mosquitoes receiving a sucrose diet was higher than in mosquitoes receiving a ZIKV-spiked blood meal on 7 dpe (Fig. 2c). Although AANV appeared to show a similar pattern as GMV, this was less pronounced, and no significant difference was found for AANV among the heads or bodies of *Ae. aegypti*, with respect to diet (Fig. 2d and Fig. S3). GCTLV genome copy numbers were very similar among the bodies of the four groups, with a median around 10^3^ copies, while they were significantly higher in the heads of mosquitoes receiving a WNV-spiked blood meal on 14 dpe (Fig. 2e), which was a pattern similar to GMV and AANV in *Aedes* (Fig. 2a and d). Interestingly, no WSLV3 genomes were detected in both body and head of mosquitoes at 14 dpe containing infectious WNV particles (except for a single sample that showed CPE in the plaque assay). In contrast, the bodies of samples without infectious WNV viral particles in the head contained significantly higher WSLV3 genome copy numbers. A similar trend was also observed in the heads, although the statistical tests were not significant (Fig. 2f). Comparable patterns were observed for the analyses of the 7 and 14 dpe analyses combined (Fig. S3).

10.1128/mbio.01021-22.4 FIG S3 Comparison of viral genome copies in body and head of mosquitoes. Pairwise Wilcoxon test: *P < *0.05 (*), *P < *0.01 (**), *P < *0.001 (***), *P < *0.0001 (****). *P* values were corrected for multiple comparisons with the Benjamini-Hochberg method. Download FIG S3, PDF file, 2.2 MB. Copyright © 2022 Shi et al. 2022 Shi et al. https://creativecommons.org/licenses/by/4.0/ This content is distributed under the terms of the Creative Commons Attribution 4.0 International license.

### Presence of ZIKV in blood meal increases bacteriome diversity profile in *Ae. aegypti*.

**FIG 3:**
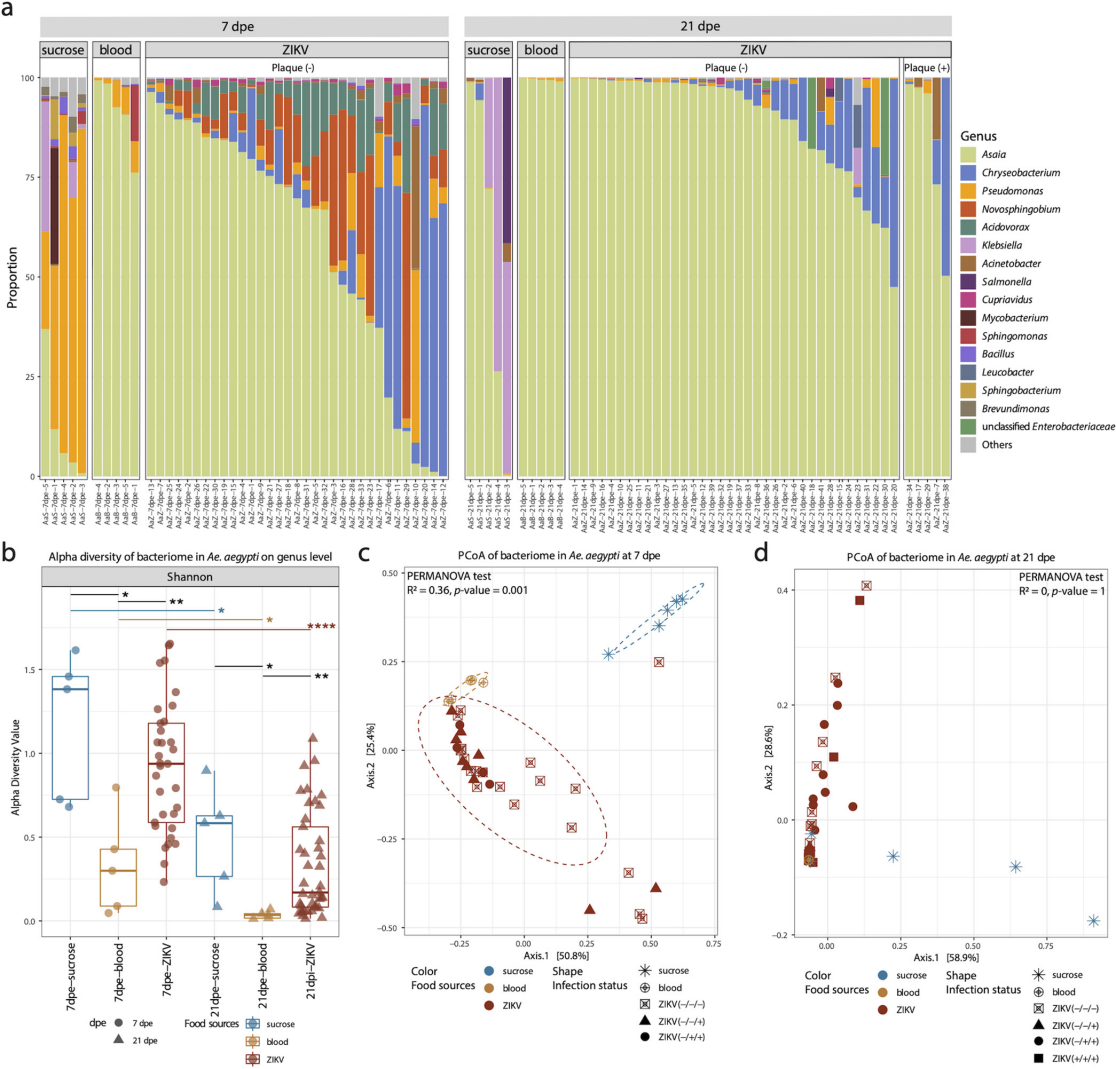
The bacteriome profile in Aedes aegypti. (a) The proportion of bacterial genera in each sample. Only the genera with total proportion above 10% are shown in the figure. (b) Alpha diversity of Aedes aegypti samples per food source group and time point on genus levels. Pairwise Wilcoxon test: *P < *0.05 (*), *P < *0.01 (**), *P < *0.001 (***), *P < *0.0001 (****). *P* values were corrected for multiple comparisons using the Benjamini-Hochberg method. (c and d) PCoA of bacterial genera based on Bray-Curtis dissimilarity on 7 dpe and 21 dpe. The PERMANOVA test was performed on food sources. The “+” or “–” in the bracket after ZIKV indicates the positive or negative result of plaque assay (head), qRT-PCR detection of ZIKV in head and body, respectively.

16S rRNA sequencing was performed on the same mosquito individual bodies to characterize their bacteriome. Overall, *Asaia* was the most prevalent genus in all *Aedes* samples receiving a blood meal with variable proportions (Fig. 3a). The bacteriome of the sucrose/water-fed group was mainly dominated by Pseudomonas at day 7, but *Asaia* and/or Klebsiella dominated after 21 days. Samples receiving a noninfectious blood meal were strongly dominated by *Asaia*, with very few other bacterial genera identified (i.e., Pseudomonas and Novosphingobium), both at 7 and 21 dpe. However, for the group fed with ZIKV-spiked blood at 7 dpe, a much higher bacterial diversity was observed (Fig. 3a). *Asaia* still dominated in most of the samples, but several samples were dominated by *Chryseobacterium*. Furthermore, *Novosphingobium* and *Acidovorax* were also frequently encountered in samples at 7 dpe. A similar observation could be made for samples at 21 dpe, although the dominance of *Asaia* was larger, followed by *Chryseobacterium* as the second most common bacterial genus. *Novosphingobium* and *Acidovorax* decreased dramatically at 21 dpe. In addition, for the mosquitoes receiving a ZIKV-spiked bloodmeal, the bacteriome profiles of infected (plaque-positive) and uninfected samples (no plaque) were undistinguishable (Fig. 3b).

The bacterial Shannon alpha diversity of the group fed with noninfectious blood was significantly lower than the group receiving sucrose/water at both time points. Interestingly, the alpha diversity of the ZIKV-spiked blood group was significantly higher than the group receiving noninfectious blood, and indistinguishable from that of the group receiving sucrose/water. For all three diet groups, the diversity decreased from 7 to 21 dpe. For the PCoA based on Bray-Curtis dissimilarity, the samples at 7 dpe largely clustered separately according to the food sources (PERMANOVA test: R^2^ = 0.36, *P = *0.001) (Fig. 3c). At 21 dpe, the mosquitoes receiving a blood meal (ZIKV spiked or not) clustered together, which overlapped with two samples of the sucrose-fed group (Fig. 3d).

### Presence of WNV in blood meal does not modify the bacteriome diversity profile in *Cx. quinquefasciatus*.

**FIG 4:**
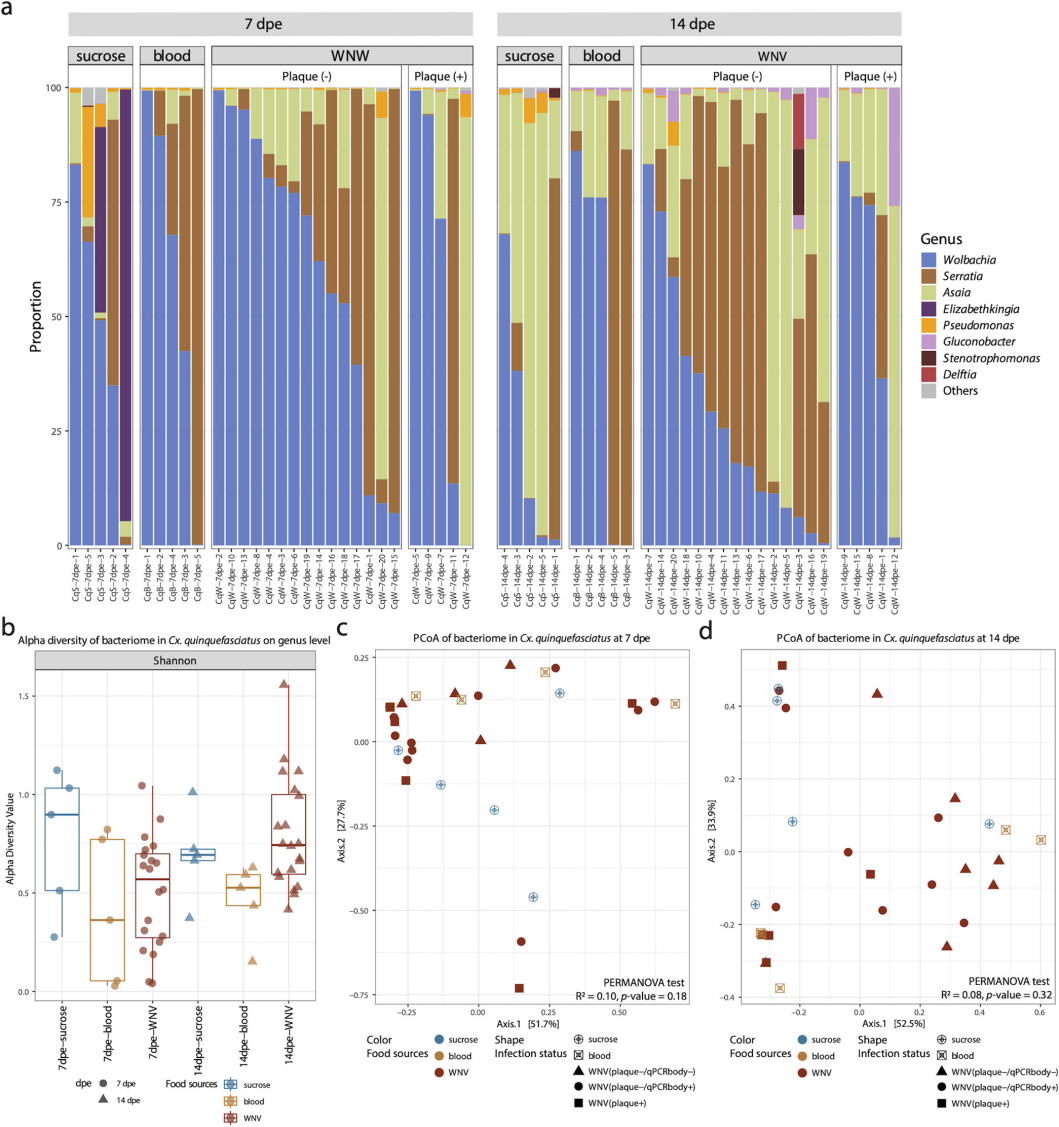
The bacteriome profile in Culex quinquefasciatus. (a) The proportion of bacterial genera in each sample. Only the genera with total proportion above 10% are shown in the figure. (b) Alpha diversity of Culex quinquefasciatus samples per food source group on genus levels. Pairwise Wilcoxon test: *P < *0.05 (*), *P < *0.01 (**), *P < *0.001 (***), *P < *0.0001 (****). *P* values were corrected for multiple comparisons using the Benjamini-Hochberg method. (c) PCoA of bacterial genera based on Bray-Curtis dissimilarity of samples on 7 dpe and 14 dpe. The PERMANOVA test was performed on food sources.

The identified members of the bacteriome in all *Culex* samples were very similar regardless of collection date and food sources, mainly occupied by *Wolbachia*, *Serratia*, and *Asaia* (Fig. 4a). Two samples in the sucrose/water group at 7 dpe contained high proportions of *Elizabethkingia*. These three diet groups showed very similar Shannon indexes of alpha diversity (Fig. 4b). Furthermore, no separation among the three diet groups was observed in the PCoA (Fig. 4c and d). However, from 7 dpe to 14 dpe, the relative abundance of *Asaia* significantly increased in all groups except for the individuals with infectious viral particles in heads (plaque +), while the abundance of *Wolbachia* decreased in the group without infectious viral particles detected (plaque –) (Fig. S4).

10.1128/mbio.01021-22.5 FIG S4 Relative abundance of *Asaia* and *Wolbachia* in Culex quinquefasciatus of two time points. Download FIG S4, PDF file, 0.1 MB. Copyright © 2022 Shi et al. 2022 Shi et al. https://creativecommons.org/licenses/by/4.0/ This content is distributed under the terms of the Creative Commons Attribution 4.0 International license.

### Differential effects of ZIKV on bacterial genera in *Aedes* mosquitoes.

**FIG 5:**
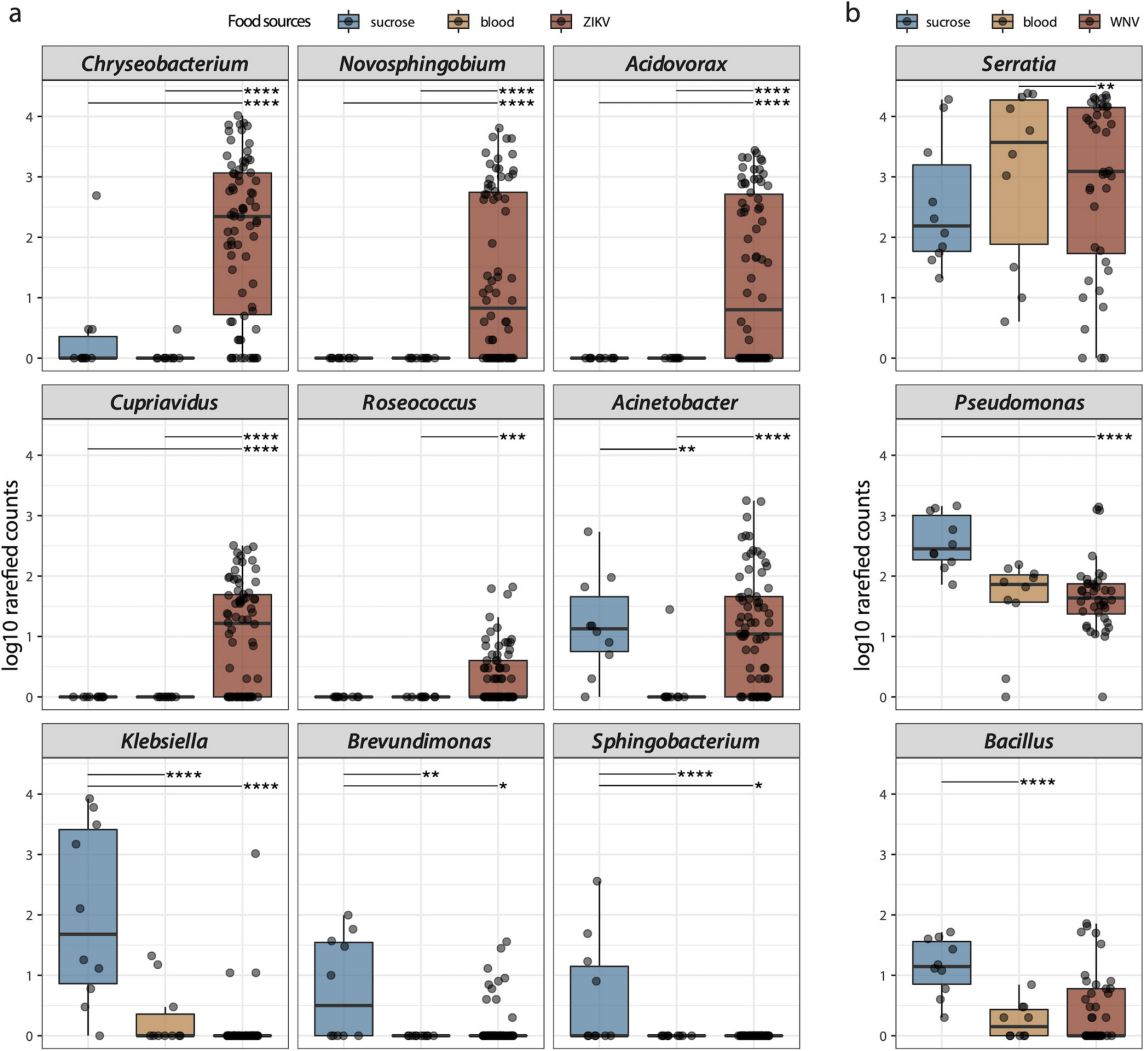
(a) The bacterial genera with differential abundance among three food source groups in Aedes aegypti. (b) The bacterial genera with differential abundance among three food source groups in Culex quinquefasciatus. These bacterial genera are determined by DESeq2 with padj <0.05 and baseMean >10 as cutoff. padj < 0.05 (*), padj < 0.01 (**), padj < 0.001 (***), padj < 0.0001 (****).

The abundance of genera *Chryseobacterium*, *Novosphingobium*, *Acidovorax*, *Cupriavidus*, and Roseococcus was higher in *Aedes* fed with ZIKV-spiked blood compared to the noninfectious blood and sucrose/water (Fig. 5a). The noninfectious blood-fed *Aedes* mosquitoes showed a decreased Acinetobacter abundance. Klebsiella, Brevundimonas, and Sphingobacterium were most abundant in the *Aedes* fed with sucrose/water and were almost absent in mosquitoes receiving a blood meal (with and without ZIKV). The noninfectious blood-fed *Culex* mosquitoes showed a higher abundance of *Serratia* compared to WNV spiked-blood-fed mosquitoes, whereas Pseudomonas and Bacillus were more abundant in sucrose/water-fed mosquitoes (Fig. 5b).

### Phageome profile in mosquito samples.

**FIG 6:**
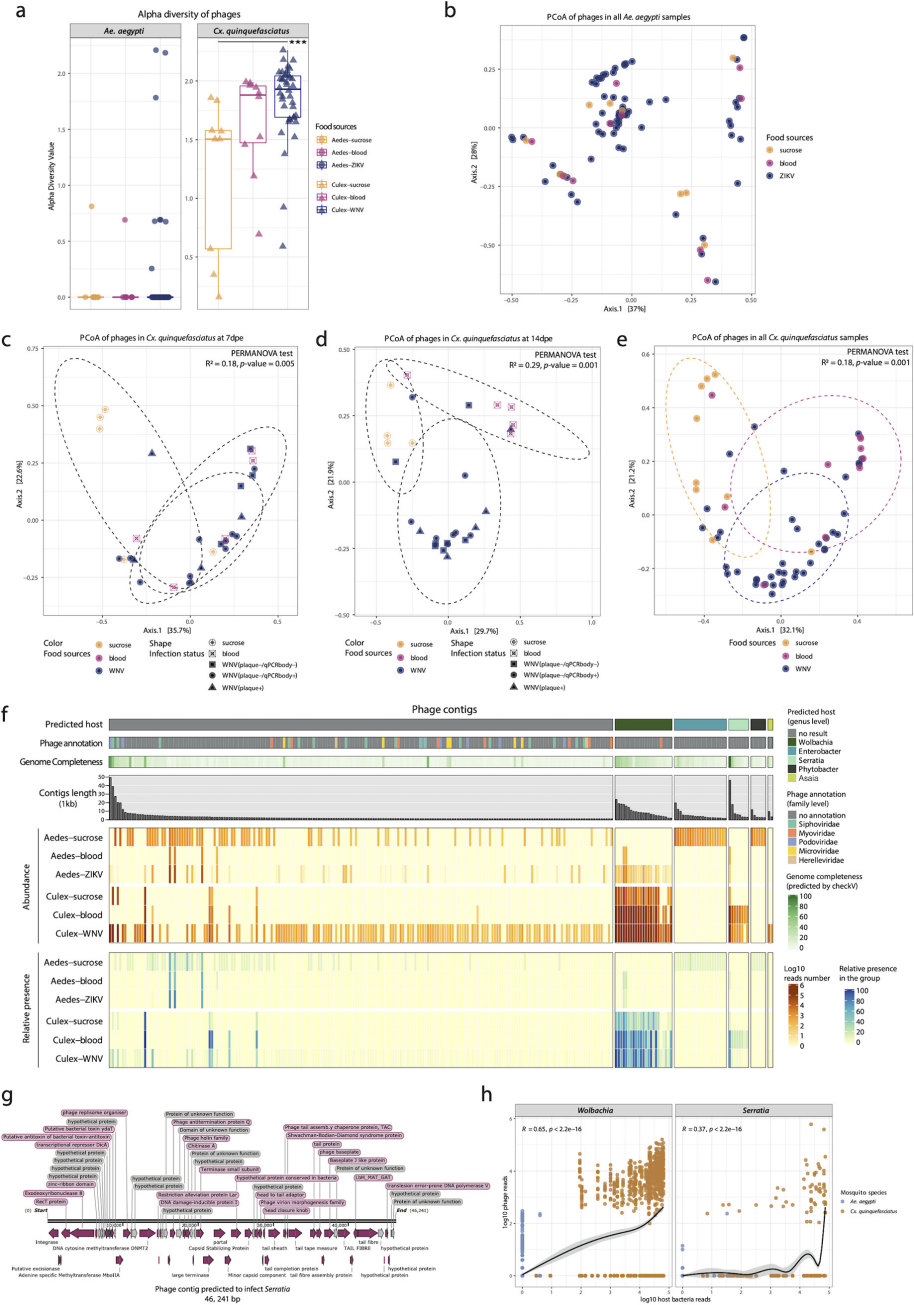
Phageome profile in mosquito samples. (a) Alpha diversity of the phageome in *Ae. aegypti* and *Cx. quinquefasciatus* on contigs with completeness of >20%. (b) PCoA of all *Ae. aegypti* samples. (c) PCoA of phageome in *Cx. quinquefasciatus* at 7 dpe. (d) PCoA of phageome in *Cx. quinquefasciatus* at 14 dpe. (e) PCoA of all *Cx. quinquefasciatus* samples. The PERMANOVA test was performed on food sources. The ellipses were drawn for each food type. (f) The profile of phage contigs. The columns are phage contigs split according to their predicted bacterial host (the top bar). The second bar indicates the taxonomic annotation of the phage contigs. The third bar is the genome completeness of the phage contigs evaluated by CheckV. The bar plot shows the length of each phage contig. The top heatmap (orangish) displays the abundance of phage contigs per food source in Aedes aegypti and Culex quinquefasciatus. The bottom heatmap (bluish) is the present proportion of each phage contig per food source. Samples containing more than 100 reads per contig are considered present. (g) Genome structure of *Serratia* phage. (h) The abundance correlation of phage contigs and their predicted host. The spearman method is used for computing correlation coefficient.

The alpha diversity (Shannon index) of the phageome in *Ae. aegypti* samples was indistinguishable among the three different diet groups (Fig. 6a). In contrast, the phageome alpha diversity in *Cx*. *quinquefasciatus* showed that mosquitoes receiving a WNV-spiked blood meal had a significantly higher diversity compared to the sucrose/water-fed mosquitoes. As could be expected, the PCoA analyses showed no separation among the three diet groups of *Ae. aegypti* (Fig. 6b). However, for the *Culex* samples, the sucrose/water-fed mosquitoes at 7 dpe, 14 dpe, or combined formed a separate cluster from the ones fed with blood (with or without WNV) (Fig. 6c to e). Contrarily to eukaryotic viruses, the diversity and abundance of phages in *Cx*. *quinquefasciatus* samples were much higher than *Ae. aegypti* (Fig. 6a and f).

For 60 out of the 263 identified phage contigs, Wolbachia, Enterobacter, Serratia, Phytobacter, or *Asaia* could be predicted to be the host (Fig. 6f and Data Set S2). Six contigs were predicted to be >50% complete (Fig. 6f and Data Set S2). Among them, one *Serratia* infecting phage genome (46,241 bp) was predicted to be 99.5% complete with high confidence. The genome organization of this phage is visualized in Fig. 6g, which contains the essential phage proteins such as tail protein, phage baseplate, and head and tail adaptor protein. Two other contigs were estimated to be 80.4% and 57.8% complete (Data Set S2) and were annotated as Podoviridae and Siphoviridae (Fig. S5). The remaining three contigs that were predicted to be more than 50% complete did not have a taxonomic annotation or predicted bacterial host (Data Set S2). In addition, the estimated completeness of the longest predicted phage contigs infecting *Wolbachia* (23,868 bp), Enterobacte (19,754 bp), *Phytobacter* (11,746 bp), and *Asaia* (9,507 bp) were 46.9%, 16.1%, 12.3%, and 17.2% complete, respectively (Data Set S2).

10.1128/mbio.01021-22.6 FIG S5 Genome structure of phage contigs. (a) Genome structure of phage contig annotated as *Podoviridae*. (b) Genome structure of phage contig annotated as *Siphoviridae*. Download FIG S5, PDF file, 0.5 MB. Copyright © 2022 Shi et al. 2022 Shi et al. https://creativecommons.org/licenses/by/4.0/ This content is distributed under the terms of the Creative Commons Attribution 4.0 International license.

10.1128/mbio.01021-22.8 DATA SET S2 Host prediction and completeness estimation of phages. Download Data Set S2, XLSX file, 0.2 MB. Copyright © 2022 Shi et al. 2022 Shi et al. https://creativecommons.org/licenses/by/4.0/ This content is distributed under the terms of the Creative Commons Attribution 4.0 International license.

For *Ae. aegypti*, distinct bacteriophage profiles could be identified between the different types of diet. Many more relatively low-abundant phage contigs could be found without known host in the sucrose-fed mosquitoes (Fig. 6f). Furthermore, a clear distinction was found between phages with a known host, with Enterobacter and *Phytobacter* phages being more prevalent in sucrose-fed mosquitoes (and absent in blood-fed mosquitoes), and *Wolbachia* infecting phages in the ZIKV-spiked blood group (which were absent in the sucrose-fed mosquitoes). For *Cx. quinquefasciatus*, the overall phage profile was rather similar between mosquitoes receiving a different diet (blood versus sucrose) and dominated by phages predicted to infect *Wolbachia*. However, the number of phage reads was higher in blood-fed mosquitoes, and the weakly abundant *Serratia* phages were largely absent in the sucrose-fed mosquitoes.

The bacteria identified by 16S rRNA and the predicted hosts of the phage contigs were largely in agreement for *Cx. quinquefasciatus*, but less so for *Ae. aegypti*. The predicted host of the phages in *Culex* samples (*Wolbachia*, *Serratia*, and *Asaia*) were found to be highly prevalent in the *Culex* bacteriome (Fig. 4a, Fig. 6f). The predicted host of the phages in *Aedes* included *Wolbachia*, Enterobacter, and *Phytobacter*. The proportion of *Wolbachia* in *Ae. aegypti* was very low (total ranging from 0.5% to 3%, and therefore not displayed in Fig. 5a) (Data Set S3). The phage contigs predicted to infect Enterobacter and *Phytobacter* were derived from a single sample AaS-21dpe-3 (Data Set S2), which contained high abundance of genera Klebsiella and Salmonella (Fig. 5a). These four genera all belong the same family *Enterobacteriaceae*. *Asaia* was the most prevalent genus present in all *Aedes* samples, but no *Asaia*-infecting phage could be identified. A possible explanation could be that 203 out of the 263 phage contigs do not have a predicted bacterial host. Potentially, the phages infecting *Asaia* in *Aedes* are too divergent to be identified using currently available approaches. In addition, the abundance of *Wolbachia* and *Serratia* positively correlated with the abundance of their respective phage contigs with *R* = 0.65 and *R* = 0.37, respectively (Fig. 6h).

10.1128/mbio.01021-22.9 DATA SET S3 16S rRNA sequencing results. Download Data Set S3, XLSX file, 0.4 MB. Copyright © 2022 Shi et al. 2022 Shi et al. https://creativecommons.org/licenses/by/4.0/ This content is distributed under the terms of the Creative Commons Attribution 4.0 International license.

## DISCUSSION

In this study, adult female mosquitoes from Guadeloupe were fed with a virus-spiked blood meal (ZIKV-spiked blood for *Ae. aegypti*, WNV-spiked blood for *Cx. quinquefasciatus*), and the infection status of their heads was determined by plaque assays as a proxy for vector competence. The observed dissemination efficiencies of ZIKV and WNV are consistent with previous studies (18, 27). The observed positivity rates of the plaque assay at 7 dpe (0% for ZIKV and 25% for WNV) (Table 1) suggest that the dissemination time of the ZIKV strain used from the midgut to the head in *Ae. aegypti* is longer compared to that of WNV in *Cx. quinquefasciatus*. The ZIKV strain used in this study was isolated in Martinique, belongs to the Asian/American lineage, and has more than 99% nucleotide identity with the ZIKV strains that circulated in both humans and mosquitoes in Guadeloupe (i.e., KX673530.1, MN185332.1). However, the transmission of this strain by *Ae. aegypti* from Guadeloupe is less efficient compared to that of ZIKV from Senegal (African lineage) due to stronger mosquito midgut infection and escape barriers (18). The vector competence of *Culex* mosquitoes from Guadeloupe has never been evaluated for WNV. Although our results show a WNV dissemination efficiency of 25%, the viral titers in the heads are rather low (from 2 to 66 PFU). Viral titers up to 10^6^ PFU per head of both insecticide-resistant and -susceptible lab mosquito lines have been reported (27). Such difference in titers suggests that *Cx. quinquefasciatus* from Guadeloupe is less susceptible to this particular WNV strain that was isolated from a horse in France (28, 29). In addition, in two mosquitoes with infectious WNV particles in their head, no WNV could be identified in the bodies by NGS or qRT-PCR (Fig. 3), suggesting that the virus might only transiently cause a midgut or systemic infection in some mosquitoes before entering the mosquito heads and salivary glands, or alternatively the virus could have gone directly from the gut to the head using the trachea network (30).

The ISVs and phages of the mosquitoes receiving different meals (virus-spiked blood, blood, or sucrose/water) were further explored. Generally, ISVs are more abundant and diverse than phages in *Ae*. *aegypti*, which is the opposite in *Cx. quinquefasciatus* (Fig. 1 and 6). This finding is consistent with the virome results from our previous study (15). The previously identified core ISVs in *Ae*. *aegypti*, such as PCLPV, GMV, ATV, and AANV, are also prevalent in samples of this study (Fig. 1a). Regarding *Cx. quinquefasciatus*, only two previously identified core eukaryotic viruses—GCTLV and PCLPV—are present in the majority of the samples as well as a novel virus annotated as WSLV3 (Fig. 1e). The *Ae. aegypti* mosquitoes receiving a blood meal showed a transiently higher ISV diversity compared to sucrose-fed animals at 7 dpe, a signal that disappeared 21 dpe (Fig. 1b). The transient nature of this change was also reflected in beta-diversity analysis (Fig. 1c and d). These observations could be explained by a major alteration or suppression of the mosquito immune response after a blood meal at 7 dpe (31), which would allow the resident ISVs to expand. Since the blood is digested 2 to 3 dpe, followed by a diet based on water and sugar, this could result in a return of the immune system to a homeostasis at 21 dpe, and a subsequent repression of ISVs. Comparable observations—correlations between changes in the host immune system and the virome—have been made in humans where immune suppressive drugs resulted in an increase in the level of anelloviruses in the blood of transplant recipients (32), or in the increase of the gut virome in nonhuman primates experimentally infected with simian immunodeficiency viruses (33). Hence, the earlier time points would be more informative to understand the effect of diet on the virome. In nature, mosquitoes undergo successive gonotrophic cycles described by getting a blood meal and laying eggs, which could result in cyclic alterations in the replication and suppression of ISV, and hence their potential vector competence for arboviruses (see above).

Since the abundance data obtained from viral metagenomics is not fully quantitative and the positive results from plaque assay need to be further confirmed, qRT-PCRs were performed to obtain the absolute load of ZIKV, WNV, and the selected core ISV genomes to better study their differential abundances among the different conditions. All selected mosquito-specific viral genomes (GMV, PCLPV, ATV, AANV, GCTLV, and WSLV3) could be detected in both head and body of most mosquitoes, sometimes reaching 10^8^ copies (Fig. 2), supporting the hypothesis that they might significantly affect arbovirus transmission. In general, mosquitoes receiving a noninfectious blood meal showed similar loads of eukaryotic viruses (PCLPV, AANV, GCTLV, WSLV3, ATV) compared to sucrose water, except for GMV, which appeared to be suppressed due to the blood meal. Some components in the blood meal may alter the immune system and therefore specifically suppress the viral replication of GMV. Interestingly, for some viruses, the presence of an arbovirus (ZIKV/WNV) in the blood meal, compared to noninfectious blood, seemed to result in an upregulation of viral genome copies (GMV, AANV, and GCTLV), although this was only strongly significant for GMV (Fig. 2a) and weakly significant in the head for GCTLV at 14 dpe (Fig. 2e). It could be hypothesized that infection by these arboviruses suppressed a specific arm of the mosquito immune system, allowing GMV and GCTLV to grow to higher titers. The associations between these ISVs and arboviruses infection or transmission need to be further investigated for confirmation and elucidating the mechanisms driving these interactions. Notably, in *Culex* samples, a statistically significant difference between the number of WSLV3 genome copies in the heads of WNV-spiked blood-fed mosquitoes was found between mosquitoes able to disseminate WNV (i.e., with infectious virus detected in heads) and mosquitoes unable to disseminate WNV (Fig. 2f). A similar trend was seen in the bodies, although not significant. This could suggest that a successful migration of WNV to the head and the saliva is inhibited by the presence of WSLV3 and that only mosquitoes without, or with very low, levels of WSLV3 are efficient to transmit WNV. If this could be confirmed, WSLV3 could be a potential bio-control agent. Similar observations have been made for the most well-studied mosquito-specific virus, Culex flavivirus (CxFV). The WNV dissemination rate in Cx. pipiens was significantly lower in a CxFV-positive colony from Colorado compared to a CxFV-negative colony from Iowa (34), but the results could vary when using different types of the mosquitoes, viruses, or infection modes (10, 13).

The identified bacterial genera in mosquitoes (Fig. 3 and 4) are similar to previous reports in *Aedes* (13, 35, 36) and *Culex* mosquitoes (37, 38). For *Ae. aegypti*, a strict sucrose diet resulted in a highly diverse bacteriome compared to mosquitoes receiving a noninfectious blood meal, in which the bacteriome was dominated by *Asaia* (Fig. 3a). In particular, Klebsiella, *Brevundimonas*, and *Sphingobacterium* were highly abundant in mosquitoes fed with sucrose solution (Fig. 5a). These results are similar to a previous study, which reported that the overall diversity of bacteria declined after a noninfectious blood meal (39). This decrease may result from the oxidative stress associated with the catabolism of the blood meal (40, 41). However, if ZIKV was present in the blood meal, a strong increase in the bacterial diversity was seen (Fig. 3a and 3b), which might again be linked to an alteration of the mosquito host immune system. This increase was mainly driven by the genera *Chryseobacterium*, *Novosphingobium*, *Acidovorax*, *Cupriavidus*, and *Roseococcus* (Fig. 5a). The enhancement of *Chryseobacterium* (Flavobacteriaceae) in mosquitoes receiving a ZIKV-spiked blood meal was in agreement with a previous study, which reported that members from the family *Flavobacteriaceae* increased early after viral exposure (3 dpe) (42). Of note, different bacteria were dominantly present in the sucrose compared to the ZIKV-spiked blood-diet mosquitoes (Fig. 3a, Fig. 5a). These observed differences became less pronounced after 21 dpe, suggesting a return to a more “normal” immune/microbiome state. In contrast, in *Cx. quinquefasciatus*, the type of meal and the WNV infection status seemed to have only little or no effect on the bacteriome composition (Fig. 4), with only Pseudomonas and *Bacillus* being increased in the sucrose/water fed mosquitoes (Fig. 5b). Furthermore, some differences were observed in the proportion of *Wolbachia*, which significantly decreased from 7 dpe to 14 dpe (for the *Culex* fed with noninfectious blood), in favor of *Asaia*, which significantly increased at 14 dpe (Fig. S4). Of interest are previous observations that *Wolbachia* has a suppressive effect on arbovirus infection, including WNV (43, 44). This might be another explanation for the low viral titer of WNV in the head of *Cx. quinquefasciatus* (Table 1). A few *Wolbachia* reads are identified in *Aedes* samples, which are likely derived from integrated *Wolbachia* sequences in the mosquito genome (45). In addition, several other bacteria are also known to interfere with host vector competence for arboviruses. For example, the *Serratia* spp. (Serratia odorifera [7] and Serratia marcescens [8]) have been reported to enhance the susceptibility of field mosquitoes to dengue virus. Pseudomonas rhodesiae isolated from the Ae. albopictus midgut show direct inhibition of La Crosse virus independent of the mosquito, which possibly produce antiviral molecules (6). Thus, it will be interesting to further explore the effects of highly abundant bacteria present in mosquitoes fed with viral blood (Fig. 5), and their potential effects on the mosquito immune response to pathogenic viruses.

Although there is no difference in the alpha and beta diversity of the phageome in *Ae*. *aegypti* (Fig. 6a and b), distinct bacteriophage profiles could be identified between the different types of diet (Fig. 6f). For *Culex* mosquitoes, the alpha diversity indices of the phageome in mosquitoes fed with WNV-spiked blood was significantly higher than those of the ones only fed with sucrose and water (Fig. 6a), which was also reflected in the separation between blood-engorged and sucrose/water-fed mosquitoes in the PCoA analyses (Fig. 6c to e). These data suggest that although the diet had little effect on the *Culex* bacteriome, the diet can induce shifts in the phage communities. However, these data should be interpreted with great caution as the majority of the identified phage genome contigs do not have a known host, nor do they represent complete genomes. For only 23% of the phage contigs, the host could be predicted. The phages infecting *Wolbachia* and *Serratia* were highly abundant and mainly present in *Culex* mosquitoes (Fig. 6f), which also showed a high abundance of their respective bacterial hosts (Fig. 4a). In correlation analysis, the abundance of phages predicted to infect *Wolbachia* and *Serratia*, and the respective bacterial host abundance, increase together, confirming the bacterial host prediction (Fig. 6h). Although it is possible that the blood is the source of bacteriophages, it is more likely that these phages are induced from prophages inside the bacterial genomes. Whether these phages are induced from dying bacteria losing the competitive battle in a changing environment, or thrive due to strong increase of their successful bacterial host, remains to be investigated.

Our single-mosquito microbiome study strategy is a promising way to further explore the interactions among the mosquito host, symbiotic microbes, and arbovirus. However, our study also had some limitations. First, the use of mosquito saliva might be a better proxy for infectivity compared to mosquito heads. Second, we cannot completely exclude that during mosquito dissection, microbes from one body part could have contaminated other body parts. Third, quantitative interpretation of metagenomics data should be done carefully, because wet lab (e.g., virus-like particle enrichment) as well as sequencing analyses could introduce biases and all data are presented as relative data.

In summary, our results reveal that different food sources and the presence of arboviruses have variable influences on the ISVs, bacteriome, and phageome interplay in *Ae. aegypti* and *Cx. quinquefasciatus*. Furthermore, our data suggest two-way interactions between specific ISVs and arboviruses. The isolation of specific ISVs and future *in vivo* infection experiments will be needed to confirm their implications in vector competence.

## MATERIALS AND METHODS

### Mosquito populations and viral strain.

Field *Ae. aegypti* at larval or pupal instar and *Cx. quinquefasciatus* imagos from Guadeloupe collected in 2018 were used for this study. *Ae. aegypti* and *Cx. quinquefasciatus* were reared by the Institut Pasteur of Guadeloupe and Institut Pasteur in Paris, respectively. The first generation (F1) of *Ae. aegypti* and *Cx. quinquefasciatus* was used in this study.

Lyophilized ZIKV strain Martinique (GenBank: KU647676) was isolated in 2015 and provided by the Emergence Virus Unit (Marseille). The WNV strain used belongs to lineage 1a and was isolated from a horse in France (Camargue) in 2000 (28). The hatched condition and virus isolation method were described in detail in Text S1.

10.1128/mbio.01021-22.1 TEXT S1 Detailed descriptions of methods. Download Text S1, DOCX file, 0.05 MB. Copyright © 2022 Shi et al. 2022 Shi et al. https://creativecommons.org/licenses/by/4.0/ This content is distributed under the terms of the Creative Commons Attribution 4.0 International license.

### Mosquito oral infections.

Seven-day-old *Ae. aegypti* and *Cx. quinquefasciatus* females were randomly separated into three groups, receiving three different diets: (i) a 10% sucrose solution; (ii) noninfectious blood meal; (iii) blood containing ZIKV (*Ae. aegypti*) or WNV (*Cx. quinquefasciatus*). *Ae. aegypti* and *Cx. quinquefasciatus* from all three groups were separately sacrificed at 7 and 21 dpe, and 7 and 14 dpe, respectively, for further analysis. More details can be found in Text S1 section “Mosquito Oral Infections.”

### Viral dissemination analysis.

Each mosquito was dissected, and the head, body, legs, and wings were collected. The heads of mosquitoes engorged with infectious blood were used to assess viral dissemination beyond the mosquito midgut by plaque assays as a proxy for the ability to transmit virus. The dissemination efficiency for ZIKV and WNV were calculated, which refers to the proportion of mosquitoes with infectious viral particles in the head forming plaques (see Text S1).

### Sample processing and sequencing for viral metagenomics and 16S rRNA sequencing.

The bodies of individual mosquitoes from all groups were homogenized with 400 μL PBS. One hundred fifty μL supernatant of each mosquito body was processed using an optimized sample preparation protocol for viral metagenomics—NetoVIR (46), as described previously. One hundred fifty-four mosquito samples (Table 2) together with 2 negative controls were sequenced on the Illumina NovaSeq 6000 high-throughput platform. Two hundred μL supernatant of each mosquito body homogenate was applied for 16S rRNA sequencing using standard lab protocols as optimized in a previous study (47). Sequencing was performed on the Illumina MiSeq platform.

### Bioinformatic analysis for viral metagenomics and 16S rRNA sequencing data.

The obtained raw paired-end reads were trimmed for quality and adapters. Reads mapping to a set of contaminating contigs known to be present in reagents were removed, and the remaining reads were *de novo* assembled. Contigs from all pools longer than 1,000 bp were clustered for redundancy. The representative contigs were taxonomically annotated using DIAMOND (48) against the nr database. All contigs annotated as eukaryotic virus were extracted using an in-house python script. Bacteriophages were identified using a combined approach with an optimized scoring system as described in Text S1. The trimmed and decontaminated reads from individual samples were mapped against the representative contigs to obtain the abundance.

After demultiplexing with sdm without allowing for mismatches, as part of the LotuS pipeline (49), 16S rRNA sequencing reads were further analyzed per sample using DADA2 pipeline (v1.6) (50) (see Text S1). Sequences annotated to the class *Chloroplast*, family mitochondria, or unknown bacteria were removed prior to the analyses. The R package decontam (51) was used to remove contaminating amplicon sequencing variants. *Aedes* mosquito samples with more than 11,428 reads were rarefied to 11,428 reads. *Culex* mosquito samples with more than 24,168 reads were rarefied to 24,168 reads. The bacterial genera with differential abundance among three food source groups were determined by R package DESeq2 with padj <0.05 and baseMean >10 as cutoff.

### Statistical analyses.

All statistical analyses were performed and visualized in R using the phyloseq (52), vegan (53), and ggplot2 (54) packages. To test median difference between two groups of continuous variables (alpha diversity measures, abundances, etc.), a pairwise Wilcoxon test was performed. Multiple testing correction was performed where appropriate using the Benjamini-Hochberg procedure (false-discovery rate [FDR] adjustment set at <0.05). Observed richness was calculated by using the phyloseq (52) package. A PERMANOVA test was used to compare beta diversity among groups.

### qRT-PCR to determine the viral genome copies in mosquito heads and bodies.

The extractions for the NGS analyses were used to determine the viral genome copies in the mosquito bodies. Viral RNA from the heads were extracted with the Qiagen Viral RNA minikit. qRT-PCRs were performed on the extractions to determine genome copies of ZIKV, PCLPV, GMV, AATV, and AANV in both head and body of *Ae. aegypti*, whereas we tested *Cx*. *quinquefasciatus* for WNV, GCTLV, and WSLV3. The reagents and analysis methods of the qRT-PCR are described in Text S1. The specific primers, probes, and qPCR conditions of each virus are in Data Set S4.

10.1128/mbio.01021-22.10 DATA SET S4 qPCR primers and probes. Download Data Set S4, XLSX file, 0.01 MB. Copyright © 2022 Shi et al. 2022 Shi et al. https://creativecommons.org/licenses/by/4.0/ This content is distributed under the terms of the Creative Commons Attribution 4.0 International license.

### Bioinformatic analysis of phageome.

The host prediction of phage contigs combined the results of multiple methods as described in Text S1, including blastn, CRISPR spacer arrays, and tRNA sequences. CheckV (55) was used to estimate the completeness of phage contigs. Only the contigs with >20% completeness were included in alpha diversity analysis. The phage contigs with >50% completeness were performed with cenote-taker2 (56) to annotate their genome structure and visualized by SnapGene software. The abundance correlation of phage contigs and their predicted bacterial host were calculated by cor.test and visualized by ggscatter in R.

### Availability of data and material.

The viral metagenomics and 16S rRNA data for this study have been deposited in the NCBI Sequence Read Archive (SRA) repository with BioProject, accession number PRJNA738559. The source data used for the statistical analysis and R script are available at https://github.com/Matthijnssenslab/MosquitoMicrobiome.
