# Supplementary material for: Bidirectional Interactions between Arboviruses and the Bacterial and Viral Microbiota in Aedes aegypti and Culex quinquefasciatus
Source: mBio. 2022 Sep 7;13(5):e01021-22. doi: 10.1128/mbio.01021-22 (PMC9600335; doi:10.1128/mbio.01021-22)
Supplement: FIG S1 [file mbio.01021-22-s0002.pdf]

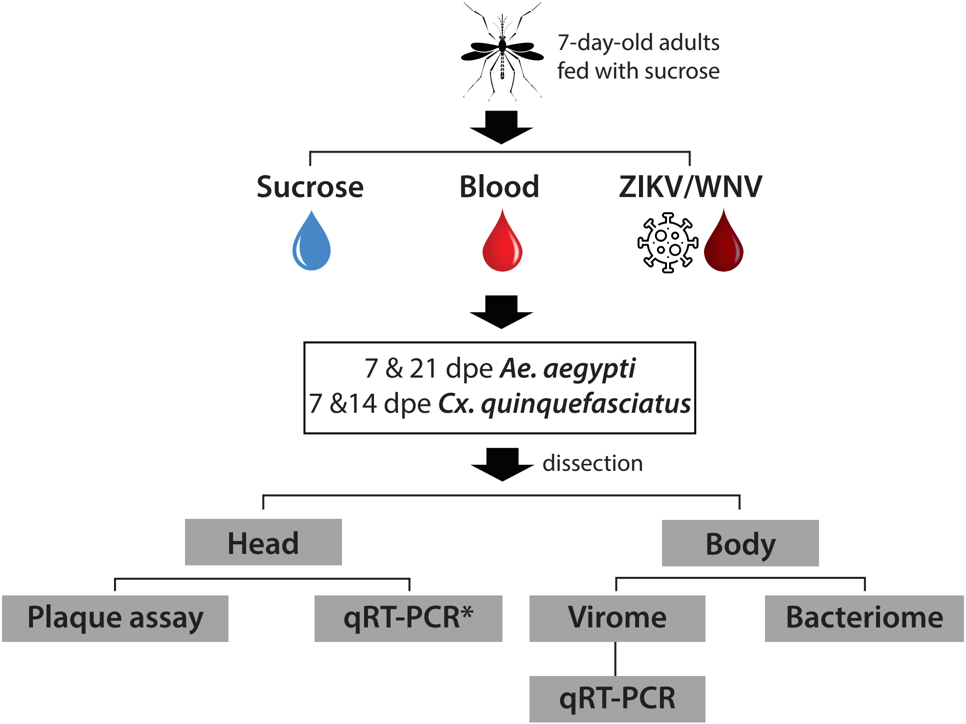
7-day-old adults
fed with sucrose
Sucrose
Blood
ZIKV/WNV
7 & 21 dpe Ae. aegypti
7 &14 dpe Cx. quinquefasciatus
dissection
Head
Body
Plaque assay
qRT-PCR*
Virome
Bacteriome
qRT-PCR
