# Supplementary figures and images for: Bidirectional Interactions between Arboviruses and the Bacterial and Viral Microbiota in Aedes aegypti and Culex quinquefasciatus
Source: mBio. 2022 Sep 7;13(5):e01021-22. doi: 10.1128/mbio.01021-22 (PMC9600335; doi:10.1128/mbio.01021-22)

# Alpha diversity of eukaryotic virus in *Ae. aegypti*

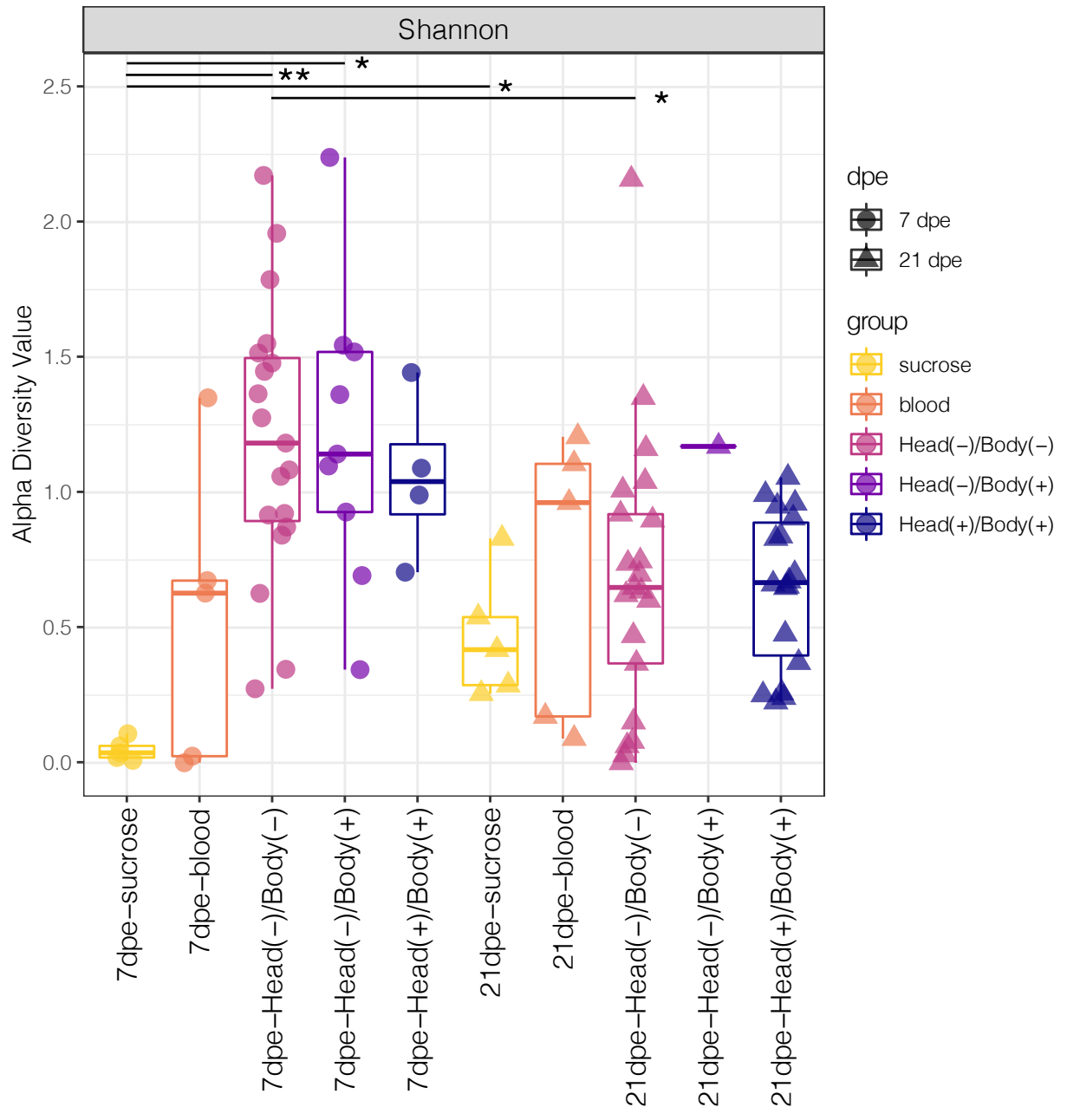

Supplement: FIG S2 [file mbio.01021-22-s0003.pdf]

Viral genome copies determined by qRT –PCR

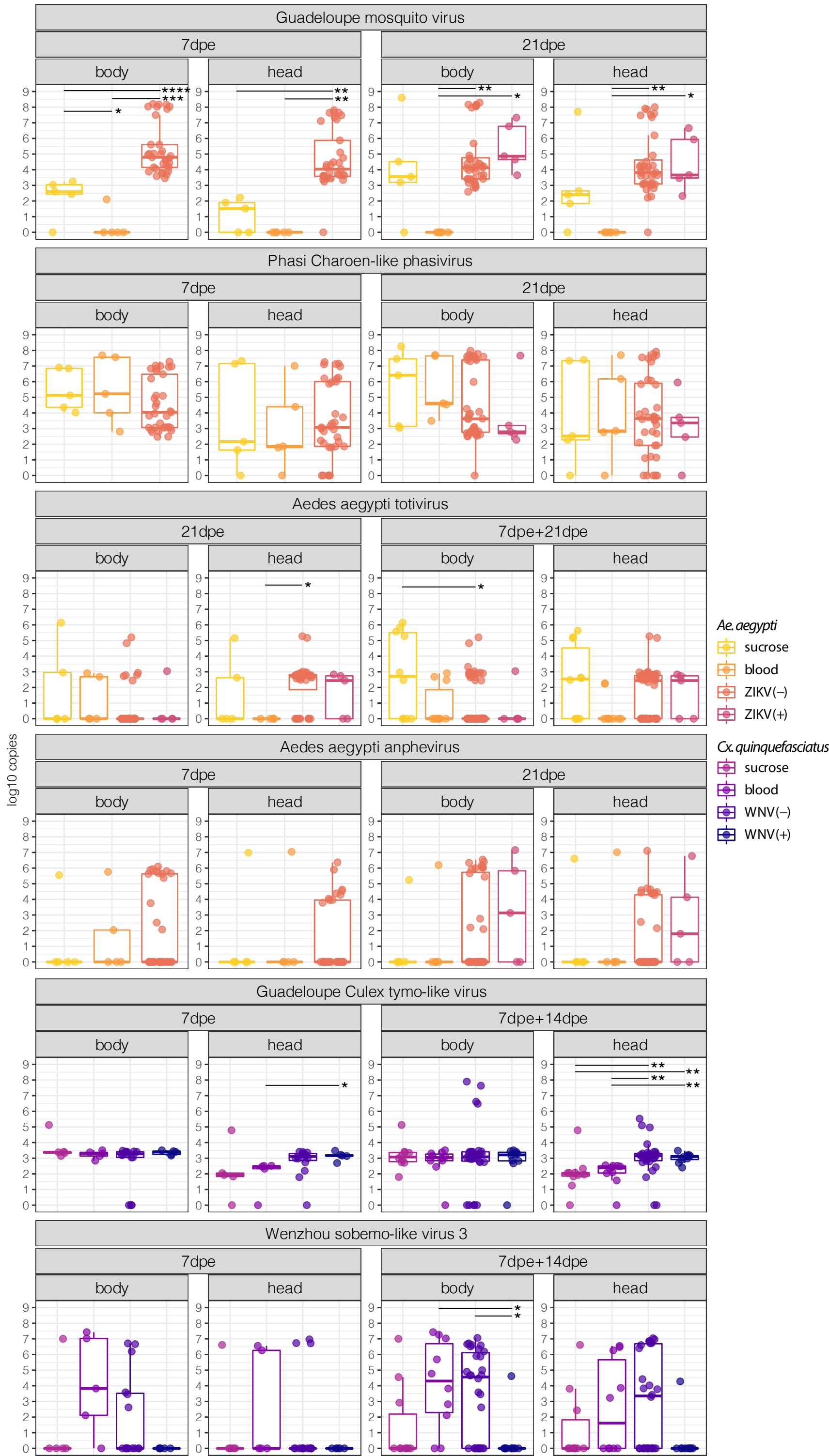

Supplement: FIG S3 [file mbio.01021-22-s0004.pdf]

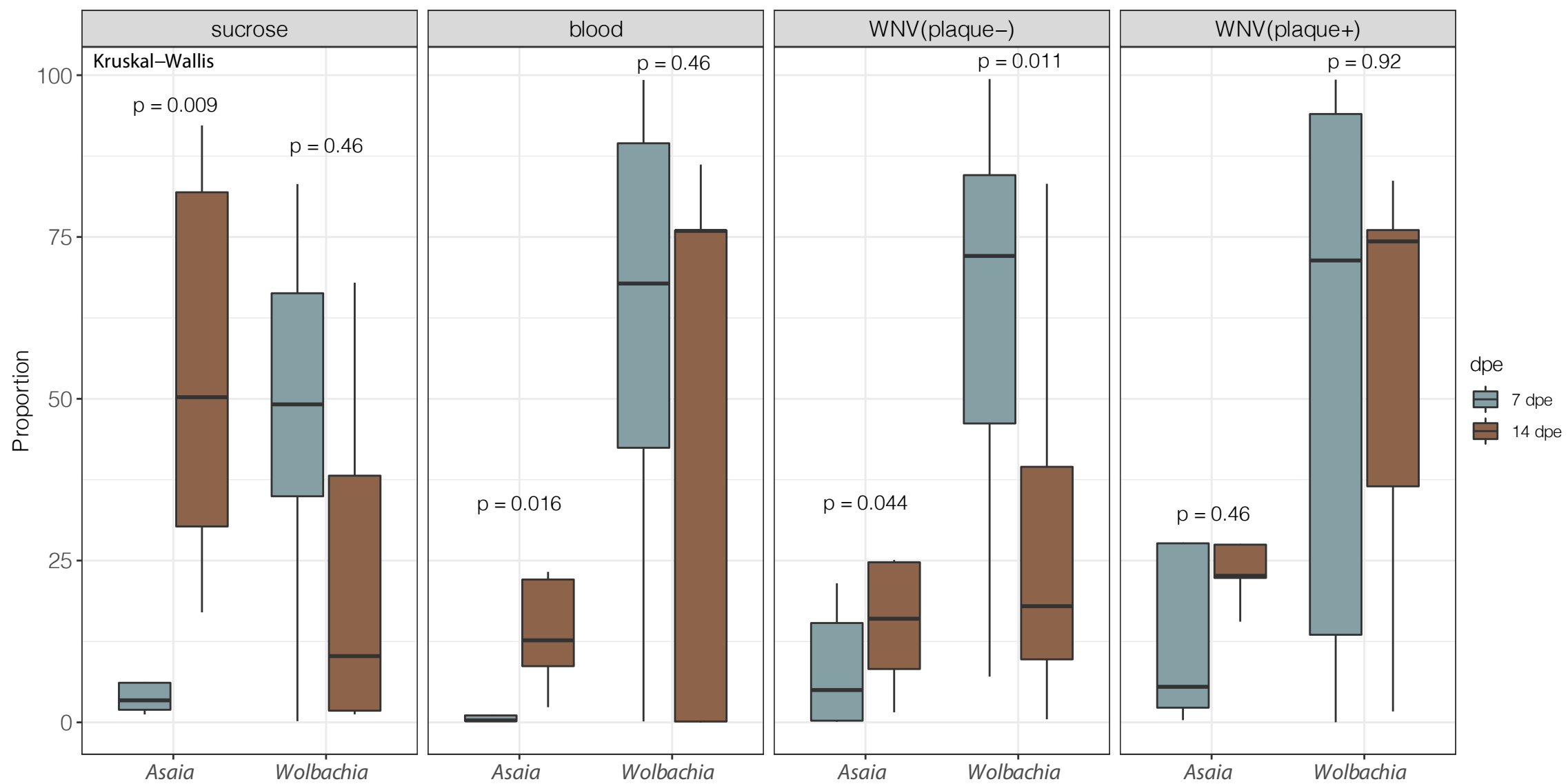

Supplement: FIG S4 [file mbio.01021-22-s0005.pdf]

a

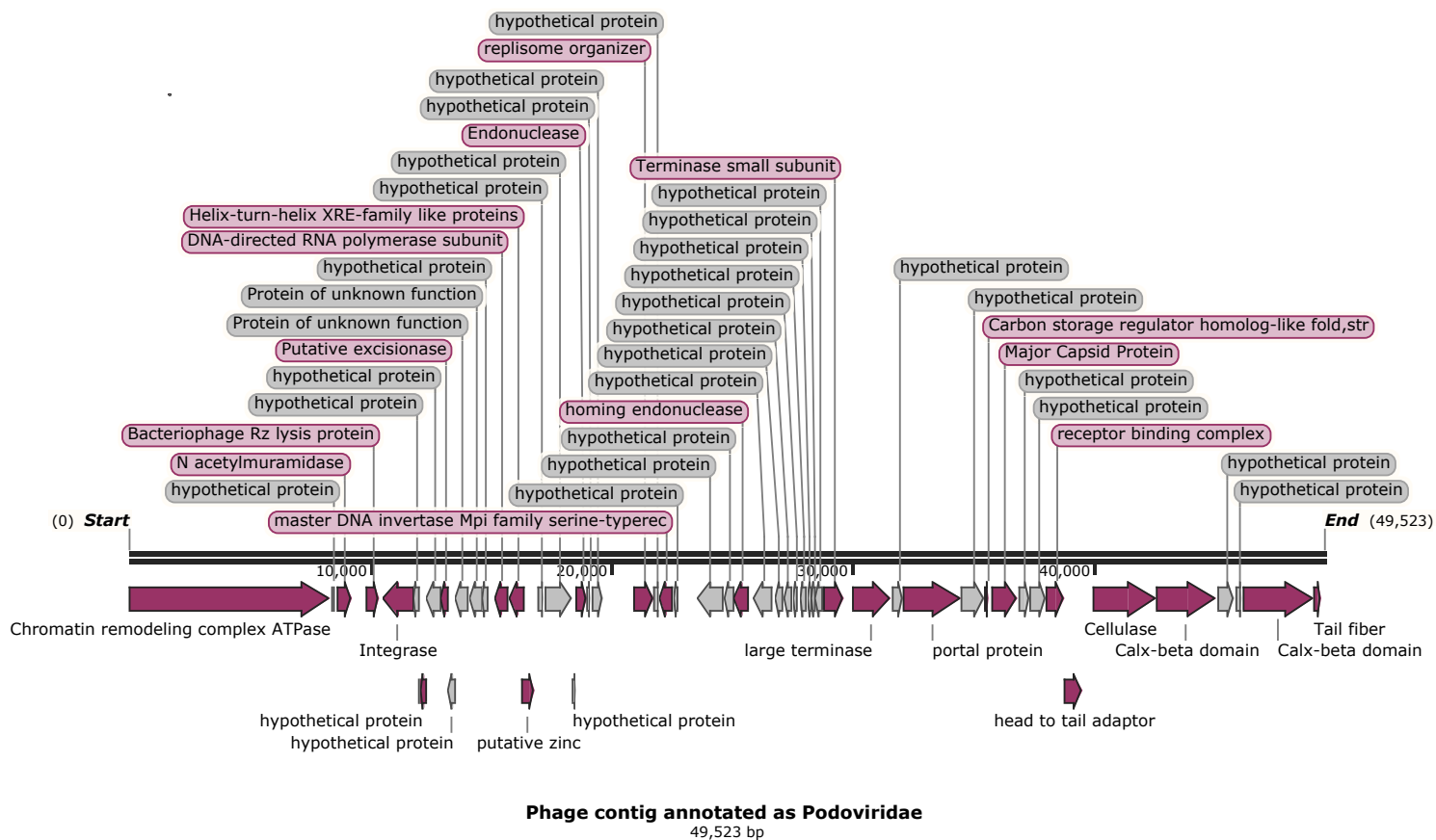

b

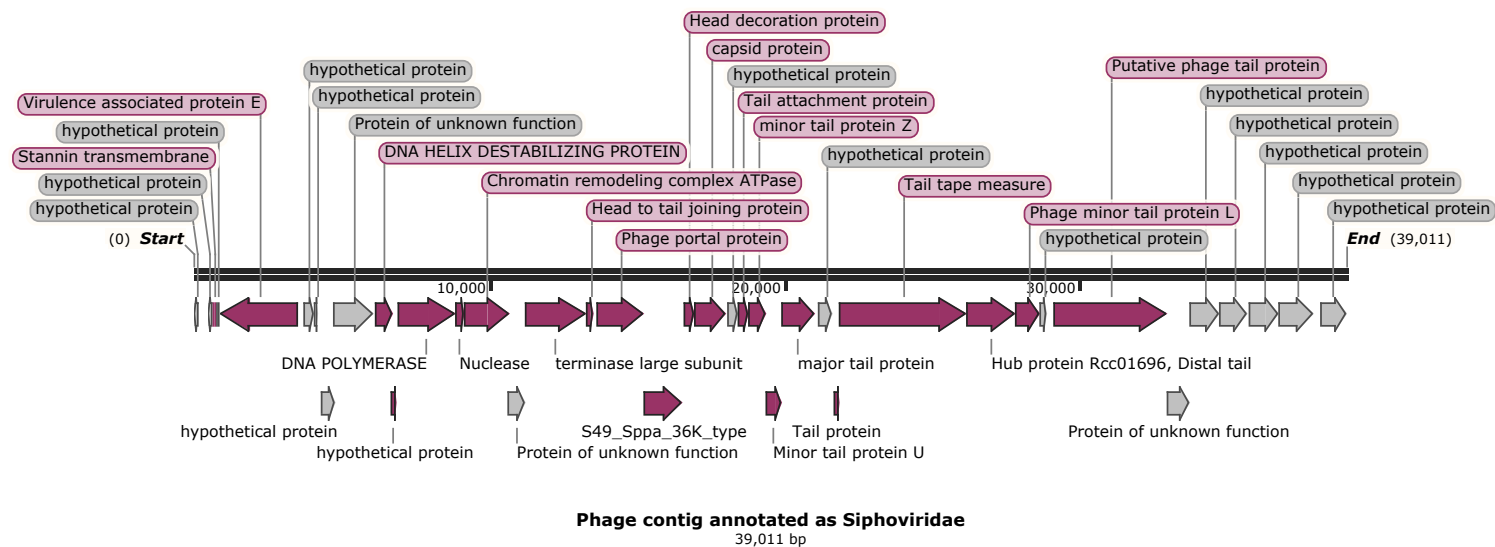

Supplement: FIG S5 [file mbio.01021-22-s0006.pdf]
