# Supplementary material for: Bidirectional Interactions between Arboviruses and the Bacterial and Viral Microbiota in Aedes aegypti and Culex quinquefasciatus
Source: mBio. 2022 Sep 7;13(5):e01021-22. doi: 10.1128/mbio.01021-22 (PMC9600335; doi:10.1128/mbio.01021-22)
Supplement: TEXT S1 [file mbio.01021-22-s0001.docx]

**Supplementary Methods**

*Ethics statement*

ZIKV oral infection experiments with *Ae. aegypti* were performed at the Institute Pasteur of Guadeloupe. Rabbit blood used for all the experiments was graciously provided by the French Agricultural Research Center for International Development (CIRAD) in Guadeloupe in the frame of a local collaboration. The animals used for blood collection are held in an Animal Experimentation Establishment (EEA) of CIRAD approved under No. C-971-18-02. This EEA was part of an ethics committee approved by the French Ministry of Education and Research. No special authorization was required in the frame of the project given the low frequency and volume of blood collections.

WNV oral infection experiments for *Cx. quinquefasciatus* were performed at the Institut Pasteur in Paris. The Institut Pasteur animal facility has received accreditation from the French Ministry of Agriculture to perform experiments on live animals in compliance with the French and European regulations on care and protection of laboratory animals. This study was approved by the Institutional Animal Care and Use Committee (IACUC) at the Institut Pasteur. No permits were required for the described field studies in locations, which are not protected in any way and did not involve endangered or protected species.

*Mosquito populations*

*Ae. aegypti* and *Cx. quinquefasciatus* from Guadeloupe collected in 2018 were used for this study. Field *Ae. aegypti* at larval or pupal instar were collected in artificial breeding-sites located in Lauricisque (16°15′01′′ N; 61°32′51′′ W), while *Cx. quinquefasciatus* imagos were collected at CIRAD (16°12′02′′ N; 61° 39′55′′ W) using BG Sentinel traps. Field collected *Cx. quinquefasciatus* were blood fed in the laboratory and egg rafts sent to the Institute Pasteur Paris to establish F1 generation. Both species were collected from 4 to 6 breeding sites. *Ae. aegypti* and *Cx. quinquefasciatus* were reared by the Institut Pasteur of Guadeloupe and Institut Pasteur in Paris, respectively. Eggs were hatched in dechlorinated tap water at 27 °C. Larvae (or pupa) were reared under controlled conditions of 150 – 200 larvae (or pupa) per 1 liter and fed with brewer’s yeast capsules renewed every 2–3 days. After adult emergence, adults were kept in flight rearing cages under controlled conditions (27 ± 1°C; 70% relative humidity; 12:12 h Light:Dark photoperiod) and fed with sterile 10% sucrose solution *ad libitum* until they were used in the experiments [1, 2]. The first generation (F1) of *Ae. aegypti* and *Cx. quinquefasciatus* was used in this study.

*Viral strain*

Lyophilized ZIKV strain Martinique (GenBank: KU647676) was isolated in 2015 and provided by the Emergence Virus Unit (Marseille) via the initiative “European Virus Archive goes global” (EVAg). Lyophilisates were re-suspended into DMEM medium (Gibco, Fisher scientific, UK) for viral production in Vero cells (ATCC, ref. CCL-81). Viral productions used a multiplicity of infection of 0.1, DMEM medium supplemented with 2% fetal bovine serum (FBS; Gibco, Fisher scientific) and were grown for three days. The ZIKV stocks obtained were kept at −80°C prior to their use in experiments. The viral titer of stocks was estimated by serial 10-fold dilutions on Vero cells and expressed as tissue culture infectious dose 50 (TCID50)/ml [1]. The WNV strain used belongs to lineage 1a and was isolated from a horse in France (Camargue) in 2000 [3]. WNV stocks were produced on *Aedes albopictus* C6/36 cells after three passages. Supernatants were harvested and stored at −80°C until experimental infections. The viral titer of stocks was determined by titration on Vero cells. For this, monolayers of Vero cells were incubated for 1 h at 37°C with serial 10-fold dilutions of supernatant. Thereafter, cells were covered with an overlay consisting of DMEM, 2% FBS, 1% antibiotic-antimycotic mix (Invitrogen, Gibco) and 1% agarose and incubated at 37°C. Cells were incubated 4 days and lytic plaques were then counted after staining with a solution of crystal violet (0.2% in 10% formaldehyde and 20% ethanol) [4]. The viral titer was expressed as PFU/ml.

*Mosquito oral infections*

Seven-day-old *Ae. aegypti* and *Cx. quinquefasciatus* females were randomly separated into three groups, receiving three different diets:

1) A 10% sucrose solution.

2) Non-infectious blood meal. Mosquitoes were allowed to feed on 2 ml of washed rabbit erythrocytes including the phagostimulant adenosine triphosphate for 1 h. Afterwards, fully engorged females were transferred in cardboard containers and maintained with 10% sucrose at 28 ± 1°C.

3) Blood containing ZIKV (*Ae. aegypti*) or WNV (*Cx. quinquefasciatus*). Infectious blood meals were prepared with ZIKV or WNV with 1.4 ml of washed rabbit erythrocytes and 700 μl of viral suspension supplemented with phagostimulant adenosine triphosphate (Sigma-Aldrich, Germany) at a final concentration of 5mM. The blood meal titer was 10^7^ TCID_50_/ml for ZIKV and 10^7^ PFU/ml for WNV. Mosquito feeding was performed with a Hemotek system and was limited to 60 min to avoid any significant decrease of blood meal titer. After the infectious blood meal, the non-engorged females were discarded, and the fully engorged mosquitoes were transferred in cardboard containers and maintained with 10% sucrose at 28° ± 1°C.

*Ae. aegypti* and *Cx. quinquefasciatus* from all three groups were separately sacrificed at 7 and 21 dpe, and 7 and 14 dpe, respectively, for further analysis.

*Viral dissemination analysis*

Each mosquito was dissected, collecting the head, body, legs and wings. The heads of mosquitoes engorged with infectious blood were used to assess viral dissemination beyond the mosquito midgut by plaque assays as a proxy for the ability to transmit virus. Heads were individually ground in 200 μl of DMEM medium supplemented with 2% fetal bovine serum (FBS) and homogenized (Precellys 24) 3 times for 20 s at 6800 rpm with 20 s pause in between using 2.8 mm ceramics beads (Precellys). The supernatant after centrifugation at 17000 g for 3 min was used for filtration with a 0.8μm pore size filter. Serial 10-fold dilutions of filtrate were incubated for 2 h at 37°C with monolayers of BHK-21 cells. Thereafter, cells were covered with an overlay of 0.8% Carboxymethyl cellulose solution containing 2% FBS and incubated at 37°C. Cells were incubated 5 days for ZIKV and 3 days for WNV and lytic plaques were then counted after staining with a solution of 1% crystal violet. The dissemination efficiency for ZIKV and WNV were calculated, which refers to the proportion of mosquitoes with infectious viral particles in the head forming plaques.

*Sample processing and sequencing for viral metagenomics*

The bodies of individual mosquitoes from all groups were homogenized (Precellys 24) with 400 µl PBS 3 times for 20 s at 6800 rpm with 20 s pause in between using 2.8 mm ceramics beads (Precellys) and then centrifuged at 17000 g for 3 min. 150 µl supernatant of each mosquito body as well as 150 µl PBS (used as negative control) was processed using an optimized sample preparation protocol for viral metagenomics – NetoVIR [5] as described previously. 154 mosquito samples (Table 2) together with 2 negative controls were sequenced on the Illumina NovaSeq 6000 High throughput platform for 300 cycles (2x150 bp paired ends).

*Bioinformatic analysis for viral metagenomics*

The obtained raw paired-end reads were trimmed for quality and adapters using Trimmomatic [6]. Reads mapping to a set of contaminating contigs known to be present in reagents and buffers were removed using BWA [7] and the remaining reads were *de novo* assembled into contigs using SPAdes [8]. Contigs from all pools longer than 1000bp were clustered for redundancy at 95% nucleotide identity over 80% of the contig length using ClusterGenomes [9]. The representative contigs were annotated using DIAMOND [10] against the nr database (updated on 29th Sep 2019) on sensitive mode for taxonomic annotation. KronaTools [11] were used to parse the output file of DIAMOND, which found the least common ancestor of the best 25 DIAMOND hits (based on BLASTx score) for each contig. The identified contigs of Kaiowa virus, Cumbaru virus, Guato virus, Chuvirus Mos8Chu0 and Trichoplusia ni TED were very likely to be endogenous viral elements from the mosquito genome as reported in previous study [12], and were removed in the analysis that followed. All contigs annotated as eukaryotic virus were extracted using an in-house python script. Bacteriophages were identified using a combined approach with an optimized scoring system. The scoring criteria are based on i) nucleotide homology – BLASTn [13] and MetaPhinder2 [14]; ii) on amino acid homology –DIAMOND and CAT [15]; iii) on genome structure – Deepvirfinder [16]; iv) ratio of gene numbers to contig length (kb); v) on gene function - eggNOG-mapper, PfamScan; vi) ratio of gene with pVOGs (Prokaryotic Virus Orthologous Groups) hit to total gene numbers in one contig; and vii) VirSorter [17].

The trimmed and decontaminated reads from individual samples were mapped (BBMap [18]) against the representative contigs of clusters that contained a contig from that samples to obtain the abundance. The eukaryotic virus and phage contigs that were present in both negative controls were removed. The obtained abundance and annotation of eukaryotic virus and bacteriophage were further used for ecological analysis in R with the phyloseq [19], vegan [20] and ggplot2 [21] packages.

*qRT-PCR to determine the viral genome copies in mosquito heads and bodies*

The extractions for the NGS analyses were used to determine the viral genome copies in the mosquito bodies. Viral RNA from the heads were extracted with the QIAGEN Viral RNA mini kit following manufacturer's instructions. qRT-PCRs were performed on the extractions to determine genome copies of ZIKV, PCLPV, GMV, AATV and AANV in both head and body of *Ae. aegypti*, whereas we tested *Cx*. *quinquefasciatus* for WNV, GCTLV and WSLV3. The specific primers and probes of each virus are in Table S4. The qRT-PCRs were run in 20 μl reaction volumes with 5 μl TaqMan Fast Virus 1-Step Master Mix (ThermoFisher), 2 μl forward and reverse primer (10 μM), 1 μl probe (5 μM), and 3 μl viral RNA extraction of samples. The standards (oligonucleotides ordered from Eurogentec and Integrated DNA Technologies) with known concentration were used to establish a calibration curve through serial tenfold dilutions (10^9^ to 10^2^ copies), subsequently used for the calculation of the viral concentration in each sample. The total copies of each virus per head or body were then calculated.

*Sample processing for 16S rRNA sequencing*

200 µl supernatant of each mosquito body homogenate were applied for 16S rRNA sequencing using standard lab protocols as optimized in previous study [22]. Briefly, DNA was extracted from mosquito samples using the RNeasy PowerMicrobiome Kit (QIAGEN), with the addition of a heating step (10min at 90°C) after vortexing/bead beating to increase DNA yield. The V4 hypervariable region of 16S rRNA genes was amplified using the 515F (GTGYCAGCMGCCGCGGTAA) and 806R (GGACTACNVGGGTWTCTAAT) primer set, modified to contain a barcode sequence between each primer and the Illumina adaptor sequences to produce dual-barcoded libraries [23]. Size selection, before Illumina sequencing, was performed using Agencourt AMPure to remove fragments below 200 bases. Sequencing was performed on the Illumina MiSeq platform (MiSeq Reagent Kit v2, 500 cycles, 15.38 % PhiX, 2*250 PE) at the VIB Nucleomics core laboratory (Leuven, Belgium).

*Bioinformatic analysis for 16S rRNA sequencing data*

After de-multiplexing with sdm without allowing for mismatches, as part of the LotuS pipeline [24], fastq sequences were further analyzed per sample using DADA2 pipeline (v1.6) [25]. In brief, after inspecting quality, sequences were trimmed to remove the primers and the first 10 bases after the primer, keeping only 200 bases and 130 for the R1 and R2 files, respectively. After merging paired sequences and removing chimeras, taxonomy was assigned using formatted SILVA training set (silva_nr_v132_train_set) [26]. Sequences annotated to the class Chloroplast, family mitochondria or unknown bacteria were removed prior to the analyses. The R package decontam [27] was used to remove contaminating Amplicon Sequencing Variants using two methods: (1) the frequency method based on the fact that contaminating sequences are expected to be inversely correlated to the input DNA concentration and (2) prevalence mode based on the assumption that contaminating sequences are prevalent in the negative control samples. *Aedes* mosquito samples with more than 11428 reads were rarefied to 11428 reads. *Culex* mosquito samples with more than 24168 reads were rarefied to 24168 reads. Comparative bacteriome analysis were performed with R packages, e.g., phyloseq [19] and DESeq2 [28]. The bacterial genera with differential abundance among three food source groups were determined by R package DESeq2 with padj < 0.05 and baseMean > 10 as cutoff.

*Bioinformatic analysis of phageome*

The host prediction of phage contigs combined the results of multiple methods. First, the bacterial genome database was built based on the phylum identified in the 16S rRNA sequencing data. Second, blastn was run for each phage contig against the bacterial genome database to detect if the identified phage contig. Third, CRISPR spacer arrays were predicted from the bacterial genomes using software MinCED [29]. The spacers were subjected to a blastn search against the phage contigs with 100 perc_identity. Lastly, the tRNA sequences were identified by tRNAscan-SE [30] from phage contigs, and blasted against the bacterial genome database with 100 perc_identity.

CheckV [31] was used to estimate the completeness of phage contigs. Only the contigs with > 20% completeness were included in alpha diversity analysis. The phage contigs with > 50% completeness were performed with cenote-taker2 [32] to annotate their genome structure and visualized by SnapGene^®^ software. The abundance correlation of phage contigs and their predicted bacterial host were calculated by cor.test and visualized by ggscatter in R.

**SI References**

1. Hery L, Boullis A, Delannay C, Vega-Rua A: **Transmission potential of African, Asian and American Zika virus strains by Aedes aegypti and Culex quinquefasciatus from Guadeloupe (French West Indies)**. *Emerg Microbes Infect* 2019, **8**(1):699-706.

2. Chouin-Carneiro T, Vega-Rua A, Vazeille M, Yebakima A, Girod R, Goindin D, Dupont-Rouzeyrol M, Lourenco-de-Oliveira R, Failloux AB: **Differential Susceptibilities of Aedes aegypti and Aedes albopictus from the Americas to Zika Virus**. *PLoS Negl Trop Dis* 2016, **10**(3):e0004543.

3. Murgue B, Murri S, Zientara S, Durand B, Durand JP, Zeller H: **West Nile outbreak in horses in southern France, 2000: the return after 35 years**. *Emerg Infect Dis* 2001, **7**(4):692-696.

4. Atyame CM, Alout H, Mousson L, Vazeille M, Diallo M, Weill M, Failloux AB: **Insecticide resistance genes affect Culex quinquefasciatus vector competence for West Nile virus**. *Proc Biol Sci* 2019, **286**(1894):20182273.

5. Conceicao-Neto N, Zeller M, Lefrere H, De Bruyn P, Beller L, Deboutte W, Yinda CK, Lavigne R, Maes P, Van Ranst M *et al*: **Modular approach to customise sample preparation procedures for viral metagenomics: a reproducible protocol for virome analysis**. *Sci Rep* 2015, **5**:16532.

6. Bolger AM, Lohse M, Usadel B: **Trimmomatic: a flexible trimmer for Illumina sequence data**. *Bioinformatics* 2014, **30**(15):2114-2120.

7. Li H, Durbin R: **Fast and accurate short read alignment with Burrows-Wheeler transform**. *Bioinformatics* 2009, **25**(14):1754-1760.

8. Bankevich A, Nurk S, Antipov D, Gurevich AA, Dvorkin M, Kulikov AS, Lesin VM, Nikolenko SI, Pham S, Prjibelski AD *et al*: **SPAdes: a new genome assembly algorithm and its applications to single-cell sequencing**. *J Comput Biol* 2012, **19**(5):455-477.

9. <https://bitbucket.org/MAVERICLab/docker-clustergenomes>

10. Buchfink B, Xie C, Huson DH: **Fast and sensitive protein alignment using DIAMOND**. *Nat Methods* 2015, **12**(1):59-60.

11. Ondov BD, Bergman NH, Phillippy AM: **Interactive metagenomic visualization in a Web browser**. *BMC Bioinformatics* 2011, **12**:385.

12. Dezordi FZ, Vasconcelos C, Rezende AM, Wallau GL: **In and Outs of Chuviridae Endogenous Viral Elements: Origin of a Potentially New Retrovirus and Signature of Ancient and Ongoing Arms Race in Mosquito Genomes**. *Front Genet* 2020, **11**:542437.

13. Altschul SF, Gish W, Miller W, Myers EW, Lipman DJ: **Basic local alignment search tool**. *J Mol Biol* 1990, **215**(3):403-410.

14. Jurtz VI, Villarroel J, Lund O, Voldby Larsen M, Nielsen M: **MetaPhinder-Identifying Bacteriophage Sequences in Metagenomic Data Sets**. *PLoS One* 2016, **11**(9):e0163111.

15. <https://github.com/dutilh/CAT>

16. <https://github.com/jessieren/DeepVirFinder>

17. Roux S, Enault F, Hurwitz BL, Sullivan MB: **VirSorter: mining viral signal from microbial genomic data**. *PeerJ* 2015, **3**:e985.

18. <https://github.com/BioInfoTools/BBMap>

19. McMurdie PJ, Holmes S: **phyloseq: an R package for reproducible interactive analysis and graphics of microbiome census data**. *PLoS One* 2013, **8**(4):e61217.

20. Dixon P: **VEGAN, a package of R functions for community ecology**. *J Veg Sci* 2003, **14**(6):927-930.

21. Wickham H: **ggplot2: Elegant Graphics for Data Analysis**. *Springer-Verlag New York* 2016.

22. Falony G, Joossens M, Vieira-Silva S, Wang J, Darzi Y, Faust K, Kurilshikov A, Bonder MJ, Valles-Colomer M, Vandeputte D *et al*: **Population-level analysis of gut microbiome variation**. *Science* 2016, **352**(6285):560-564.

23. Tito RY, Chaffron S, Caenepeel C, Lima-Mendez G, Wang J, Vieira-Silva S, Falony G, Hildebrand F, Darzi Y, Rymenans L *et al*: **Population-level analysis of Blastocystis subtype prevalence and variation in the human gut microbiota**. *Gut* 2019, **68**(7):1180-1189.

24. Hildebrand F, Tadeo R, Voigt AY, Bork P, Raes J: **LotuS: an efficient and user-friendly OTU processing pipeline**. *Microbiome* 2014, **2**(1):30.

25. Callahan BJ, McMurdie PJ, Rosen MJ, Han AW, Johnson AJ, Holmes SP: **DADA2: High-resolution sample inference from Illumina amplicon data**. *Nat Methods* 2016, **13**(7):581-583.

26. Quast C, Pruesse E, Yilmaz P, Gerken J, Schweer T, Yarza P, Peplies J, Glockner FO: **The SILVA ribosomal RNA gene database project: improved data processing and web-based tools**. *Nucleic Acids Res* 2013, **41**(Database issue):D590-596.

27. Callahan BJ, Davis NM: **decontam: Identify Contaminants in Marker-gene and Metagenomics Sequencing Data**. 2019.

28. Love MI, Huber W, Anders S: **Moderated estimation of fold change and dispersion for RNA-seq data with DESeq2**. *Genome Biol* 2014, **15**(12):550.

29. <https://github.com/ctSkennerton/minced>**.**

30. Chan PP, Lowe TM: **tRNAscan-SE: Searching for tRNA Genes in Genomic Sequences**. *Methods Mol Biol* 2019, **1962**:1-14.

31. Nayfach S, Camargo AP, Schulz F, Eloe-Fadrosh E, Roux S, Kyrpides NC: **CheckV assesses the quality and completeness of metagenome-assembled viral genomes**. *Nat Biotechnol* 2020.

32. Tisza MJ, Belford AK, Dominguez-Huerta G, Bolduc B, Buck CB: **Cenote-Taker 2 democratizes virus discovery and sequence annotation**. *Virus Evol* 2021, **7**(1):veaa100.
